# New perspectives on YTHDF2 O-GlcNAc modification in the pathogenesis of intervertebral disc degeneration

**DOI:** 10.1186/s10020-024-00876-x

**Published:** 2024-10-18

**Authors:** Liangjie Lu, Lijun Wang, Minjie Yang, Huihan Wang

**Affiliations:** 1 Department of Orthopedics, Ningbo Medical Center Li Huili Hospital, Li Huili Hospital, Affiliated to Ningbo University, No.57 Xingning Road, Yinzhou District, Ningbo, 315040 Zhejiang Province China; 2 Department of Pediatrics, The First Hospital of Jilin University, Changchun, 130021 China; 3 Department of Orthopaedics, Jiu jiang NO.1 People’s Hospital, Jiu jiang, 332000 China; 4 Department of Orthopaedics, Zhengzhou Central Hospital Affiliated to Zhengzhou University, Zhengzhou, 450007 China

**Keywords:** Intervertebral disc degeneration, O-GlcNAc modification, YTHDF2, CCNE1, Cell cycle, Oxidative stress

## Abstract

This study investigates the potential molecular mechanisms by which O-GlcNAc modification of YTHDF2 regulates the cell cycle and participates in intervertebral disc degeneration (IDD). We employed transcriptome sequencing to identify genes involved in IDD and utilized bioinformatics analysis to predict key disease-related genes. In vitro mechanistic validation was performed using mouse nucleus pulposus (NP) cells. Changes in reactive oxygen species (ROS) and cell cycle were assessed through flow cytometry and CCK-8 assays. An IDD mouse model was also established for in vivo mechanistic validation, with changes in IDD severity measured using X-rays and immunohistochemical staining. Bioinformatics analysis revealed differential expression of YTHDF2 in NP cells of normal and IDD mice, suggesting its potential as a diagnostic gene for IDD. In vitro cell experiments demonstrated that YTHDF2 expression and O-GlcNAcylation were reduced in NP cells under H_2_O_2_ induction, leading to inhibition of the cell cycle through decreased stability of CCNE1 mRNA. Further, in vivo animal experiments confirmed a decrease in YTHDF2 expression and O-GlcNAcylation in IDD mice, while overexpression or increased O-GlcNAcylation of YTHDF2 promoted CCNE1 protein expression, thereby alleviating IDD pathology. YTHDF2 inhibits its degradation through O-GlcNAc modification, promoting the stability of CCNE1 mRNA and the cell cycle to prevent IDD formation.

**Supplementary Information:**

The online version contains supplementary material available at 10.1186/s10020-024-00876-x.

## Introduction

Intervertebral disc degeneration (IDD) is a prevalent orthopedic condition closely associated with symptoms like lower back pain in daily activities, which greatly affects the quality of life for patients (Francisco et al. 2022). The development of IDD is the outcome of intricate interactions involving molecules and cellular processes (des Portes 2020). Despite extensive research, the precise molecular mechanisms underlying IDD remain incompletely understood (Lv et al. 2022). An in-depth study of IDD not only helps in understanding the etiology and progression of the disease, but it may also offer novel insights and strategies for its treatment (Ramírez et al. 2023).

In recent years, there has been considerable interest in protein modification, particularly O-GlcNAc modification (Chatham et al. 2021). O-GlcNAc modification is a dynamic and reversible protein glycosylation process widely distributed among organisms (Xue et al. 2022). It governs numerous cellular physiological and pathological processes by influencing proteins’ stability, activity, and interactions (Anand et al. 2022; Xiao et al. 2023). Aberrant O-GlcNAc modification may be implicated in numerous human diseases, including intellectual and developmental disabilities (IDD) (Chatham et al. 2021; Liu et al. 2022; Saha et al. 2021).

Epigenetic modifications, particularly RNA modifications, have been demonstrated to play crucial roles in numerous biological processes, including cell fate determination, cell differentiation, and stress responses. N6-methyladenosine (m6A) is the most prevalent endogenous mRNA modification, and it has been shown to exert significant influence in various biological processes (Meyer and Jaffrey 2014). The functions of m6A are primarily mediated through its “writers,” “erasers,” and “readers” proteins. These proteins comprise writers such as METTL3, METTL14, and WTAP, erasers including FTO and ALKBH5, and readers like YTHDF1, YTHDF2, YTHDF3, YTHDC1, and YTHDC2 (Wang et al. 2014). YTHDF2, a recently identified RNA-binding protein, plays a crucial role in various biological processes, specifically in regulating the stability and degradation of RNA (Yang et al. 2022; Zhang et al. 2019). Furthermore, YTHDF2 has been discovered to accept O-GlcNAc modification, introducing additional complexity to its cellular physiology and pathology (Yang et al. 2023a). Initial research suggests that the O-GlcNAc modification of YTHDF2 may be associated with regulating RNA targets and participating in cellular processes (Sarris et al. 2020).

In summary, understanding the molecular mechanism of IDD continues to present a challenge. However, the protein YTHDF2 and its modification through O-GlcNAc may play a crucial role in this process (Shao et al. 2021). Considering the significance of YTHDF2 in the cell cycle, protein synthesis, and degradation, this study seeks to investigate the precise role of O-GlcNAc modification in regulating the cell cycle of YTHDF2, thereby impacting the development and progression of IDD. Through in-depth research on YTHDF2, I aim to identify novel molecular targets for the diagnosis and treatment of IDD.

Our research findings indicate a significant differential expression of YTHDF2 in NP cells of normal and intervertebral disc degeneration (IDD) mice, suggesting its potential as a diagnostic gene for IDD. In vitro experiments revealed a notable reduction in both the expression of YTHDF2 and its O-GlcNAcylation (O-GlcNAc modification) in NP cells induced by H_2_O_2_, leading to the inhibition of cell cycle progression via decreased stability of CCNE1 mRNA. Further, in vivo experiments confirmed a substantial decrease in the expression of YTHDF2 and its O-GlcNAc modification in IDD mice, while overexpression or increased O-GlcNAc modification enhanced the expression of CCNE1 protein, thereby ameliorating the severity of IDD. In summary, our study demonstrates that YTHDF2, through O-GlcNAc modification, influences the stability of CCNE1 mRNA and cell cycle regulation, providing novel therapeutic strategies for IDD.

## Materials and methods

### High-throughput transcriptome sample sequencing

Transcriptome sequencing samples were collected from C57BL/6 N mice subjected to tail suspension for 4 weeks to induce IDD. Control groups consisted of normal mice, with 8 mice in each group. We collected nucleus pulposus (NP) cells from the intervertebral discs of mice for high-throughput transcriptome sequencing analysis (RNA-seq). For differential analysis, the transcriptome sequencing results were compared between the experimental group (IDD group mice) and the control group (normal mice).

Total RNA was extracted from each sample using Trizol reagent (ThermoFisher, 15,596,026, USA). RNA concentration, purity, and integrity were determined using the Qubit^®^2.0 Fluorometer^®^ (Life Technologies, Q33216, USA) with the Qubit^®^RNA Analysis Kit (Shanghai Boji Biotechnology Co., Ltd., HKR2106-01, Shanghai, China), the Nanometer Spectrophotometer (IMPLEN, USA), and the RNA Nano 6000 Analysis Kit of the Bioanalyzer 2100 System (Agilent, 5067 − 1511, USA), respectively. Each sample contains a total RNA content of 3 µg, utilized as the input material for RNA sample preparation.

Per the manufacturer’s recommendation, the NEBNext^®^UltraTM RNA Library Prep Kit (NEB, E7435L, Beijing, China), specifically designed for Illumina^®^ (USA), should generate cDNA libraries. The quality of these libraries should then be assessed using the Agilent Bioanalyzer 2100 system. The indexed-encoded samples were clustered on the cBot cluster generation system using the TruSeq PE Cluster Kit v3 cBot HS (Illumina) (PE-401-3001, Illumina, USA), following the manufacturer’s instructions. Following cluster generation, library preparation was performed and sequenced on the Illumina HiSeq 550 platform, producing paired-end reads with a length of 125 bp/150 bp.

Quality check was conducted on the paired-end reads of the raw sequencing data using FastQC software v0.11.8. The raw data was preprocessed using Cutadapt software version 1.18 to eliminate Illumina sequencing adapters and poly(A) tail sequences. A perl script was employed to eliminate reads with N content surpassing 5%. Reads with a quality score greater than 20, comprising 70% of the total base quality, were extracted using FASTX Toolkit software version 0.0.13. Repairing paired-end sequences using BBMap software. Lastly, the high-quality filtered read fragments were aligned to the mice’s reference genome using hisat2 software (version 0.7.12) (Arunachalam et al. 2022; Linkner et al. 2021).

### Bioinformatics methods

Screening for differentially expressed mRNAs using the ‘limma’ package in the R language. Volcano plots could be generated using the ggplot2 package in R, while heatmaps could be created using the pheatmap package. Draw a Venn diagram using the jvenn database (http://jvenn.toulouse.inra.fr/app/example.html).

Plotting ROC curve using the “pROC” package. The ssGSEA analysis was conducted using the software packages “GSEABase” and “GSVA” (Liang et al. 2022; Du et al., 2022a).

#### Mouse NP cell isolation and culture

We chose three male C57BL/6 N mice aged 4–6 weeks and obtained their nucleus pulposus (NP) tissue from the lumbar vertebrae segments L3 ∼ L5. Subsequently, the collected tissue was placed in culture dishes filled with DMEM cell culture medium (Gibco, 11,965,126). The NP tissue has been cut into small pieces using sterile tools like scissors and microtome knives. For the digestion mixture preparation, collagenase XI (SIGMA, Sigma-C7657) and protease II (Merck, 42613-33-2) were combined in the correct proportions and then added to the cell culture medium. Transfer the excised tissue fragments into a petri dish filled with cell culture medium supplemented with collagenase XI and protease II.

The petri dish was placed in an incubator set at 37 °C overnight to enable the tissue to be digested by enzymes and for cells to be released. The following morning, the digested tissue mixture was transferred into a sterile centrifuge tube after removing the petri dish. The cells were then sedimated at the bottom of the centrifuge tube through low-speed centrifugation, and the supernatant was subsequently discarded. Following removing the supernatant, the cell pellet was resuspended in a DMEM cell culture medium to guarantee homogeneous cell dispersion. Next, the cell suspension was transferred to a cell culture medium comprising 2% penicillin/streptomycin and 10% fetal bovine serum.

Under normal conditions, the culture dish is usually placed in a cell culture incubator (51,026,537, Thermo Fisher, USA) set at 37℃, with 5% CO2 and saturated humidity, for cultivation. To count cells, fully digest them using trypsin, halt the digestion process and achieve even cell dispersion by adding a complete medium supplemented with 10% fetal bovine serum. The cell sample (10 µL) was mixed with 0.4% trypan blue solution (10 µL), and then 10 µL of the resulting mixture was added to a Countess cell counting slide (C10228, Invitrogen, USA). The count was obtained using a Countess cell counter (AMQAX2000, Invitrogen, USA). The plating and subsequent experiments are conducted based on the cell count.

The cells were divided into 6 groups and named as follows: the NC group (no treatment), the H_2_O_2_ group (NP cells treated with H_2_O_2_), the sh-NC + OE-NC group (NP cells treated with silencing control and overexpression control), the H_2_O_2_ + sh-NC + OE-NC group (NP cells treated with H_2_O_2_ and with silencing control and overexpression control), the H_2_O_2_ + sh-NC + YTHDF2 group (NP cells treated with H_2_O_2_ and with silencing control and YTHDF2 overexpression), and the H_2_O_2_ + sh-YTHDF2 + OE-NC group (NP cells treated with H_2_O_2_ and with YTHDF2 silencing and overexpression controls). Cells were treated with 100 µM of H_2_O_2_ or PBS for 24 h and transfected with different plasmids using lentivirus.

In this experiment, we treated NP cells with a 20 µM OGA inhibitor TMG (Thiamet G, HY-12,588, MCE) or a 25 µM OGT inhibitor OSMI-1 (HY-119,738, MCE) to investigate the regulation of CCNE1 mRNA stability by YTHDF2 O-GlcNAc. Additionally, we performed gene overexpression and knockdown through lentivirus transduction while inhibiting transcription using streptolydigin (5 µg/mL, HY-17,559, MCE).

Additional reagents, including MG132 (HY-13,259), chloroquine (CQ, HY-17,589 A), and bafilomycin A1 (BafA1, HY-100,558), were also obtained from MCE company (Yang et al. 2023b; Che et al. 2020).

### Lentivirus infection

To establish infection with lentiviruses, it is necessary to seed the target cells into cell culture dishes or 6-well plates 24 h before infection. A preferable cell density of 50% was achieved on the second day of infection. The target and control cells were infected separately with the target vector virus solution. After 24 h, the old culture medium was replaced with fresh medium and further cultivation was carried out for 48 h before conducting subsequent experiments.

The lentiviral vector is pCDH-CMV-MCS-EF1-GFP + Puro (Changsha Aibibiotechnology, HG-VMS0751). The shRNA plasmid vector used in this study is pLKO.1, while the plasmid and lentivirus packaging services were provided by Guangzhou Sengong Biotechnology, located in Guangzhou, China. The sequences are as follows: sh-YTHDF2: 5’-GCAAACTTGCAGTTTATGTAT-3’; sh-OGT: 5’-GCAGCTTATCTTCGTGCCTTA-3’; sh-OGA: 5’-TATACTATCAGACCTTATTTC-3’; sh-CCNE1: 5’-CCATATTGTCAGAACAGAATA-3’; sh-NC sequence: 5’-CCTAAGGTTAAGTCGCCCTCG-3’. The plasmid constructed with the luciferase reporter gene was co-transfected into 293T cells (ATCC, CRL-3216) with the helper plasmid. After verification, amplification, and purification, the lentivirus was successfully packaged.

5 × 10^5^ cells were seeded in a 6-well plate for lentivirus-mediated cell infection. Once the cells reached 70–90% confluency, they were infected with an appropriate amount of packaged lentivirus (MOI = 10, working titer approximately 5 × 10^6^ TU/mL) and 5 µg/mL polystyrene sulfonate (Merck, TR-1003, USA) in the culture medium. After 4 h of infection, an equal volume of culture medium was added to dilute the polystyrene sulfonate. After 24 h of infection, a fresh culture medium was substituted. After 48 h of infection, the infection status was observed using a luciferase reporter gene, and stably transfected cell lines were obtained by treating with an appropriate concentration of puromycin (A1113803, Gibco, Grand Island, NY, USA) for resistance selection. Cells were collected when they no longer underwent cell death in the medium containing the purine analog, and the knockdown efficiency was confirmed using RT-qPCR (Li et al. 2021).

### Establishment of intervertebral disc degeneration model in mice (IDD)

Male C57BL/6 N mice (4–6 weeks old, purchased from Beijing Vital River Laboratory Animal Technology Co., Ltd., Beijing, China) were housed in a 24-hour light/dark cycle at 25 °C. After one week of acclimatization, the mice were randomly divided into 4 groups: sh-NC + OE-NC (treated with silencing control and overexpression control), IDD + sh-NC + OE-NC (IDD model group, treated with silencing control and overexpression control), IDD + sh-NC + YTHDF2 (IDD model group, treated with silencing control and YTHDF2 overexpression), and IDD + sh-YTHDF2 + OE-NC (IDD model group, treated with YTHDF2 silencing and overexpression controls), each group consisting of 6 mice.

To establish a mouse IDD model, all groups of mice, except the control group, underwent tail suspension for 4 weeks to induce IDD (Figure S1). During this period, the mice were housed in specialized cages where their tails were suspended while having free access to food and water. During the breeding period, mice were manipulated to achieve gene overexpression and knockdown by intradiscal injection of lentivirus into the nucleus pulposus. The lentivirus had a multiplicity of infection (MOI) of 10, with a working titer of approximately 1 × 10^9^ transducing units (TU) per milliliter, administered every 4 days.

In the second round of animal experiments, 24 IDD mice were divided into groups as follows: shControl (transfection with a control silencing vector), shYTHDF2 (transfection with YTHDF2 silencing vector), shYTHDF2 + WT (transfection with YTHDF2 silencing vector alongside overexpression of Flag-YTHDF2 wild type), and shYTHDF2 + S263A (transfection with YTHDF2 silencing vector alongside overexpression of Flag-YTHDF2-S263A mutation where S263A represents a serine to alanine mutation at position 263 of YTHDF2). In the third round, 36 IDD mice were divided into shControl (control silencing vector), shCCNE1 (CCNE1 silencing vector), shYTHDF2 (YTHDF2 silencing vector), shYTHDF2 + OE-CCNE1 (YTHDF2 silencing vector alongside overexpression of CCNE1), shYTHDF2 + OE-CCNE1 + WT (YTHDF2 silencing vector alongside overexpression of CCNE1 and Flag-YTHDF2 wild type), and shYTHDF2 + OE-CCNE1 + S263A (YTHDF2 silencing vector alongside overexpression of CCNE1 and Flag-YTHDF2-S263A mutation). The mice in each group were treated differently only by injection of different lentiviruses. In the fourth round, 12 IDD mice were divided into IDD and IDD + TMG groups, where the IDD + TMG group received TMG injections intraperitoneally every 4 days, while the IDD group received an equivalent volume of saline. Prior to euthanizing the animals, X-ray images were taken to measure the disc height index (DHI) by calculating the average values of the posterior, middle, and anterior parts of the intervertebral disc based on the vertebral body measurements collected from the X-rays.

The lumbar vertebrae (L3-L6) from mice were decalcified for four days following fixation in a 14% paraformaldehyde (PFA) solution (Merck Life Science, 30525-89-4). Nanoparticle organizations utilize paraffin embedding and sectioning techniques to acquire 5-µm-thick slices for histological staining or immunohistochemistry (IHC) purposes. To evaluate intervertebral disc degeneration, paraffin sections were treated with dewaxing hydration and stained with hematoxylin and eosin (H&E) to examine the cellular and tissue morphology. Safranin O staining was performed to analyze the proteoglycan content. Senescent cells were identified using age-related β-galactosidase (SA-β-gal) staining. The experimental protocol and procedures for animal use have received approval from the Institutional Animal Ethics Committee (Ji et al. 2012; Kiviranta et al. 1985; Wang et al. 2009).

### H&E staining

The paraffin sections of mouse NP tissue were sequentially treated as follows: xylene I for 10 min, xylene II for 10 min, anhydrous ethyl alcohol I for 5 min, anhydrous ethyl alcohol II for 5 min, 95% ethanol for 5 min, 90% ethanol for 5 min, 80% ethanol for 5 min, 70% ethanol for 5 min, followed by a rinse with distilled water. After dewaxing and dehydrating the sections, they were immersed in Harris hematoxylin for 3–8 min, then rinsed with tap water, differentiated in 1% hydrochloric acid ethanol for a few seconds, rinsed with tap water again, and finally blue in 0.6% ammonia water before being rinsed with running water. The slice was stained in the eosin dye solution for 1–3 min. The slices were immersed in 95% ethanol I for 5 min, followed by 95% ethanol II for 5 min, anhydrous ethanol I for 5 min, anhydrous ethanol II for 5 min, xylene I for 5 min, and xylene II for 5 min to achieve dehydration and clarification. Afterwards, the slices were removed from the xylene and allowed to air dry slightly before being mounted on neutral resin slides. Microscope inspection (Nikon, TE200). All the experimental reagents mentioned above were obtained from Sangon Biotech (Guangzhou, China) (Li et al. 2018).

#### Immunohistochemistry (IHC)

The tissue sections underwent antigen retrieval treatment with 10 mM sodium citrate at 100 °C. Additionally, the sections were treated with H_2_O_2_ in 10% PBS to deactivate the endogenous peroxidase. Next, the slices were blocked using 10% goat serum and incubated overnight at 4 °C with the following antibodies: β-galactosidase (ab203749, Abcam; 1:200), 8-hydroxy-2’-deoxyguanosine (8-OHdG) (ab48508, Abcam; 1:200), Ki67 (ab15580, Abcam; 1:200), PCNA (ab92552, Abcam; 1:200), YTHDF2 (ab246514, Abcam; 1:2000), and CCNE1 (MA5-14336, Invitrogen; 1:200). Next, the slices were treated with biotinylated goat anti-mouse or anti-rabbit IgG. Subsequently, they were incubated with the ABC Peroxidase Standard Staining Kit (32,020, Thermo Scientific) for 30 min. We conducted staining using 3,3’-diaminobenzidine and then performed counterstaining with hematoxylin. The percentage of positive cells is determined by calculating the ratio between the number of cell nuclei positively labeled and the total number of cells labeled with hemoglobin (Yukata et al. 2018).

### Safranin staining

The sliced tissues were placed in 70% ethanol for dewaxing treatment. Rinse tissue sections with distilled or deionized water to eliminate any remaining wax and impurities. The sliced tissue was soaked in an acidic alcoholic phenol solution for 1 to 3 min. Gently rinse the tissue sections with distilled water to remove any remaining acidic alcohol. Per the instructions, the sliced tissue was placed into the Safranin O staining solution (G1067, Cell Signaling Technology) for 2–5 min. Gently rinse tissue slices with distilled water until the dye ceases to leak. The dehydration treatment involved a series of ethanol solutions with progressively higher concentrations (starting from 70% ethanol, then 95% ethanol, and finally absolute ethanol). Each concentration was used to treat the specimens for several minutes. The sliced tissue sections were placed into a mixture containing differential stain and clearing agent, with the permeation time adjusted as necessary. The sliced tissue was placed on a slide and fixed in an oven (Kiviranta et al. 1985).

### qRT-PCR

Tissue and whole-cell RNA were extracted using Trizol (catalog number 16,096,020, Thermo Fisher Scientific, New York, USA). To obtain cDNA, reverse transcription was performed using the reverse transcription reagent kit (RR047A, Takara, Japan). The reaction system was prepared using the SYBR^®^ Premix Ex TaqTM II kit (DRR081, Takara, Japan). The qRT-PCR reaction was performed on a real-time fluorescence quantitative PCR instrument (ABI 7500, ABI, Foster City, CA, USA). The program consisted of an initial incubation at 95 °C for 10 min, followed by 35 cycles of incubation at 95 °C for 15 s, 60 °C for 30 s, and 72 °C for 45 s. GAPDH serves as an internal reference gene. qRT-PCR settings were performed in triplicate wells and repeated three times. The value of 2^-ΔΔCt^ represents the fold change in the expression level of the target gene between the experimental group and the control group. The formula for calculating ΔΔCT is ΔΔCT = ΔCt experimental group - ΔCt control group, where ΔCt is the difference in Ct values between the target gene and the reference gene. Ct represents the number of amplification cycles necessary for the real-time fluorescence intensity to reach a predetermined threshold, indicating that the amplification is in the logarithmic phase of growth. The primer information used in the experiment is presented in Table S1 (Wang et al. 2022).

### Western blot

The cells were lysed using RIPA lysis buffer (P0013B, Beyotime Biotechnology, Shanghai, China), which was supplemented with 1% protease inhibitor and phosphatase inhibitor. The separation of nucleus/cytoplasmic proteins was carried out following the protocol provided in the kit (ab219177, Abcam). The total protein concentration was quantitatively measured using the BCA method and the BCA assay kit (A53226, Thermo Fisher Scientific, Rockford, IL, USA). Following separating proteins by SDS-PAGE electrophoresis, we employed the wet transfer method to transfer the proteins onto a PVDF membrane (IPVH85R, Millipore, MA, USA). The membrane was incubated in a 5% BSA solution at room temperature for 1 h and then incubated with the primary antibody overnight at 4℃. The detailed antibody information is shown in (Table S2).

The membrane was washed three times with TBST, with each wash lasting 5 min. Subsequently, the membrane was incubated with HRP-conjugated secondary antibody IgG (ab6721, 1:5000, Abcam) at room temperature for 2 h. The membrane was washed three times for 5 min each using Tris-buffered saline with Tween-20 (TBST). After washing, the membrane was treated with an ECL detection reagent and analyzed using a chemiluminescence analyzer. Protein quantification analysis was conducted using ImageJ 1.48u software (V1.48, National Institutes of Health, USA) by calculating the ratio of grayscale intensity between each protein and the reference protein (Yu et al. 2020). Repeat the experiment three times each time.

### Flow Cytometry

Total production of reactive oxygen species (ROS), proliferation of NP cells, and progression of cell cycle were evaluated using 2,7-dichlorodihydrofluorescein diacetate (Sigma Aldrich, 4091-99-0), propidium iodide staining (Sigma Aldrich, 25535-16-4), and the EdU Flow Cytometry Assay Kit (Invitrogen, C10418). A single-cell suspension of mouse neural progenitor cells (NPCs) was prepared in a phosphate-buffered solution. Following treatment with specific reagents, the cells were incubated at 37℃ for 30 min. Subsequently, the cells were centrifuged to obtain cell pellets. Lastly, the FACSCalibur flow cytometer was used to conduct a flow cytometry analysis of the samples (Adan et al. 2017).

### CCK-8

The CCK-8 assay kit (C0037, Beyotime) was used for the assessment of cell proliferation. The cells were inoculated into a 96-well plate, treated with different groups, 10 µL of CCK-8 solution was added to each well, and then incubated for 1 h. Finally, the absorbance was measured at a wavelength of 450 nm using an enzyme-linked immunosorbent assay (ELISA) reader. The CCK-8 assay kit (C0037, Beyotime) is utilized for the assessment of cell proliferation. Inoculate cells into a 96-well plate, treat cells with different groups, add 10 µL of CCK-8 solution to each well, and incubate for 1 h. Finally, the absorbance was measured at a wavelength of 450 nm by utilizing an enzyme-linked immunosorbent assay (ELISA) reader (Chen et al. 2020).

### YTHDF2 O-GlcNAc site prediction

The Flag-YTHDF2 plasmid was transfected into the cells once the density of NP cells reached 70–80%. The transfected cells were collected and washed with PBS. The cells were then lysed using a cell lysis buffer containing a protease inhibitor. The cell lysate was collected by centrifugation to remove cellular debris. The buffer for cell lysis had the Flag antibody added to it and was incubated overnight at 4 °C with gentle shaking. Protein A/G magnetic beads were added to the mixture, which was then shaken and incubated at 4 °C for 2–3 h. Magnetic separation was employed to isolate the magnetic beads, which were subsequently washed to eliminate non-specifically bound proteins.

The immunoprecipitated samples were electrophoresed on an SDS-PAGE gel and the gel was stained with Coomassie Brilliant Blue dye to visualize the protein bands. Gel strips corresponding to the expected size of YTHDF2. The sliced gel strips were then sent to Shanghai Zhongke New Life Biotechnology Co., Ltd. for liquid chromatography-tandem mass spectrometry (LC-MS/MS) analysis. The mass spectrometry data was analyzed to identify the O-GlcNAc sites of YTHDF2, with expected potential sites including Ser262, Ser263, and Thr524 (Peng et al. 2017; Hahne et al. 2013).

### RNA immunoprecipitation (RIP)

RNA immunoprecipitation was conducted using the Magna RIP™ RNA-Binding Protein Immunoprecipitation Kit (17–700, Meck) per the manufacturer’s instructions. Cell lysis was achieved using RIP lysis buffer, followed by freeze-thaw treatment. Following centrifugation, the supernatant was incubated with protein A/G magnetic beads conjugated with either anti-Flag or anti-YTHDF2 antibodies for 4 to 6 h. Subsequently, the immunoprecipitate was washed and treated with Proteinase K, followed by RNA extraction. The relative interaction between YTHDF2 and the target RNA was determined using RT-qPCR and then normalized to the input (Yang et al. 2023a).

### YTHDF2 protein Stability Assay

To evaluate the stability of endogenous YTHDF2 protein, NP cells were treated with 100 µM cycloheximide (CHX, HY-12,320, MCE) and subsequently harvested at 0, 12, 24, and 36-hour time points. To evaluate the stability of exogenous YTHDF2 protein (wild type and S263A mutant), we expressed Flag-WT or Flag-S263A mutants ectopically in NP-shYTHDF2 cells. Subsequently, cells were collected at 0, 12, 24, and 36 h after treatment with 100 µM CHX. To facilitate comparisons, we normalized the Flag-YTHDF2 expression levels (WT or S263A) to a similar level at the beginning (0 h) (Li et al. 2021).

### Immunoprecipitation (IP)

After overexpressing Flag-YTHDF2 in the cells, they were harvested using a lysis buffer containing 1× protease inhibitor (P8340, Roche) and 1× phosphatase inhibitor (P1081, Beyotime Biotechnology) (10,812,846,001, 71,383, E9884, 329-98-6, L3771, 9036-19-5, Sigma) in 50 mM Tris-HCl (pH 7.4), 1 mM EDTA, 150 mM NaCl, and 1% Triton X-100. Following centrifugation, the supernatant was incubated overnight at 4 °C with anti-FLAG M2 affinity gel (A2220, Sigma-Aldrich). To perform co-immunoprecipitation, cells were co-transfected with full-length OGT and YTHDF2 proteins labeled with HA or Flag tags. The pretreated cell lysate was incubated with an anti-HA antibody overnight at 4 °C and then incubated with Protein A/G Sepharose beads (LSKMAGAG02, MCE) for 4 h. The immunoprecipitates were washed and analyzed by immunoblotting using the corresponding antibodies (Zheng et al. 2020).

### Ubiquitination experiment in cells

The cells were transfected with HA-ubiquitin (HA-Ub) or co-transfected with HA-ubiquitin and Flag-tagged YTHDF2 (WT or S263A). To prevent degradation, 10 µM of MG132 (133407-82-6, Merck Life Science) was added before collection. Forty-eight hours post-transfection, cells were harvested using a lysis buffer containing 1% SDS. The samples were subsequently boiled to denature the proteins. Immunoprecipitation was then performed utilizing either anti-Flag or anti-YTHDF2 antibodies, followed by incubating the supernatant with Protein A/G agarose beads for 4 h. Afterward, the captured complex was washed four times before being subjected to Western blot analysis. The ubiquitination level of YTHDF2 was detected using anti-K48 and anti-HA antibodies (Deng et al. 2020).

### Extracellular ubiquitination analysis

Initially, Flag-tagged YTHDF2 (WT or S263A) was transiently transfected into NP cells to purify different YTHDF2 proteins employing anti-FLAG M2 affinity gel. Subsequently, the plasmids encoding HA-FBW7, Myc-Cullin-1, Myc-SKP1, and Myc-RBX1 were transfected into HEK293 cells. The SCFFBW7 E3 ligase complex was then purified from the whole cell lysate. Next, the Flag-YTHDF2/SCFFBW7 complex, purified His-OGT, together with E1, E2, ubiquitin, ATP, and UDP-GlcNAc (HY-112,174, MCE), was be incubated in a reaction buffer (20 mM Tris-HCl, pH 7.4, 5 mM MgCl2, 1 mM DTT) following the instructions provided by the manufacturer (Abcam, ab139469). Incubate the reaction at 37 °C for 2 h, followed by termination using 2×SDS-PAGE buffer. Finally, the complex was subjected to immunoblotting using an anti-YTHDF2 antibody to detect the ubiquitination of YTHDF2 (Inuzuka et al. 2011; Popov et al. 2010).

### sWGA pull-down experiment

Nucleoprotein complexes and cells were lysed using a lysis buffer that included protease and phosphatase inhibitors (125 mM NaCl, 50 mM Tris pH 7.4, 5 mM EDTA, and 0.1% NP-40). The upper clear liquid is treated with a denaturation buffer containing sugar and protein to denature its contents. Afterward, it is digested with PNGase (P0704S, New England Biolabs) to remove N-linked glycoproteins. Following centrifugation, the supernatant was incubated overnight at 4℃ with agarose beads (AL-1023 S, Vector Laboratories, Burlingame) conjugated to sWGA. The precipitated complex underwent washing and was subsequently subjected to immunoblotting using anti-YTHDF2 or anti-Flag antibodies. All input samples displayed were adjusted to comparable levels in the subsequent sWGA binding experiments (Hoshida et al. 1999).

### Immunofluorescence staining (IF)

The cell slides were fixed in 4% paraformaldehyde for 25 min and incubated overnight with primary antibodies, including anti-YTHDF2 (ab246514, Abcam, 1:50) or anti-OGT (ab96718, Abcam, 1:50). Next, the slides were incubated with secondary antibodies labeled with fluorescence, and nuclear staining was conducted using DAPI (BioVision, S0001). The obtained images were captured with a Leica confocal microscope (Leica TCS SP8, Leica Microsystems, Wetzlar, Germany) (Yang et al. 2013).

#### Enzyme-linked immunosorbent assay (ELISA)

ELISA assays were conducted to detect proteins IL-1β (Beyotime, PI301), IL-6 (Beyotime, PI326), and TNF-α (Beyotime, PT512). Taking IL-1β ELISA as an example, the experimental steps involved preparing cell culture supernatants under different treatment conditions. This included coating plates with mouse IL-1β antibody, adding mouse IL-1β standard, biotinylated mouse IL-1β antibody, streptavidin-HRP, washing solution, color development solution, and stop solution. The number of coated plates needed for each experiment was calculated and placed in a 96-well frame. Samples were diluted with sample dilution solution at ratios of 1:5, 1:10, 1:50, 1:100, 1:1000, and 1:2000 to determine the optimal detection concentration. A protein standard curve for IL-1β was created, with working concentrations set at 1000 pg/mL, 500 pg/mL, 250 pg/mL, 125 pg/mL, 62.5 pg/mL, and 31.25 pg/mL. Samples, standard solutions, and diluents were added at 100 µL per well to their respective groups for testing, standard and blank. Biotinylated antibodies were added at 100 µL per well to all wells, covered with a plate seal, and incubated for 1 h at room temperature in the dark. After washing the plate five times with 300–400 µL of washing solution per well, with the final wash dried with absorbent paper, samples were incubated again with streptavidin-HRP at 100 µL per well under similar conditions. Following another round of washing, TMB color development solution was added at 100 µL per well, covered, and incubated for 30 min at room temperature in the dark. The reaction was stopped by adding 50 µL of stop solution, mixed, and A450 values were immediately measured. The average absorbance values of duplicate standard and sample groups were calculated. A standard curve for IL-1β was plotted with standard concentrations on the x-axis and A450 values on the y-axis, connecting the points with a smooth line. The sample concentrations were calculated using the sample absorbance values and the standard curve (Zhang et al. 2020).

### Statistical analysis

All data were processed using SPSS 21.0 statistical software. The continuous variables were reported as mean ± standard deviation. The two groups were compared using a t-test, while the comparison among multiple groups was analyzed using one-way ANOVA. A p-value less than 0.05 indicates a difference between the means of the two sample groups.

## Results

### Role of m6A-related genes in intervertebral disc degeneration: identification of YTHDF2 as a potential diagnostic biomarker and its correlation with immune cell infiltration and cell cycle pathways

Despite research on the roles played by these m6A-related genes in various biological processes, their functions and potential mechanisms in IDD remain unclear. Thus, acquiring a more profound comprehension of the role of m6A-related genes in IDD could offer novel strategies and targets for preventing and treating IDD.

**Fig. 1:**
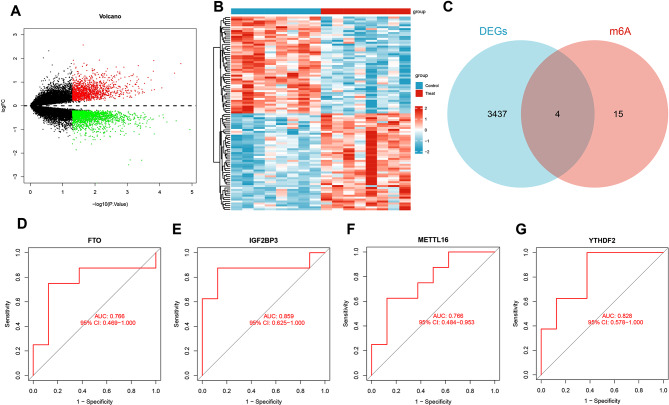
Identification of diagnostic genes for intervertebral disc degeneration through bioinformatics analysis. Note: **(A)** Heatmap and volcano plot of differentially expressed genes between the control group sample (Control group, normal mice, *n* = 8) and the treatment group sample (Treat group, IDD group, *n* = 8), where red dots represent upregulated genes, green dots represent downregulated genes, and black dots represent genes with no significant difference between the two groups; **(B)** Heatmap of the top 50 differentially expressed genes between the control group sample and the experimental group sample; **(C)** Venn diagram showing the intersection between differentially expressed genes and m6A genes; **(D)** ROC curve of FTO as a diagnostic gene for the disease; **(E)** ROC curve of IGF2BP3 as a diagnostic gene for the disease; **(F)** ROC curve of METTL16 as a diagnostic gene for the disease; **(G)** ROC curve of YTHDF2 as a diagnostic gene for the disease

In order to investigate the involvement of m6A-related genes in the progression of intervertebral disc degeneration (IDD) and identify diagnostic genes for IDD, we utilized C57BL/6 N mice as experimental subjects, dividing them into two groups of 8 mice each. The control group consisted of normal mice, while the experimental group underwent tail suspension for 4 weeks to induce IDD (Figure S1). After four weeks, we extracted nucleus pulposus (NP) tissue from the lumbar intervertebral discs of the mice for high-throughput transcriptome sequencing analysis (RNAseq). Using the control group as the reference group and the IDD group as the treatment group, we conducted differential analysis on the transcriptome sequencing results, identifying a total of 3441 differentially expressed genes (DEGs), including 1380 upregulated genes and 2061 downregulated genes. These findings were visualized in a volcano plot (Fig. 1A).

Furthermore, we have chosen the top 50 genes that show differences in upregulation and downregulation and represented them in a heat map (Fig. 1B). We conducted an intersection analysis between the 3441 differentially expressed genes (DEGs) and the set of 19 m6A-related genes, as illustrated in the Venn diagram. This analysis identified four candidate genes: FTO, IGF2BP3, METTL16, and YTHDF2 (Fig. 1C).

Next, we assessed the accuracy of these four candidate genes in diagnosing IDD by using ROC curves. The results demonstrated that the genes FTO (AUC = 0.766), IGF2BP3 (AUC = 0.859), MLKL (AUC = 0.766), and YTHDF2 (AUC = 0.828) had an area under the curve (AUC) values of 0.766, 0.859, 0.766, and 0.828, respectively. It suggests that these four genes could be considered diagnostic biomarkers for IDD with a certain level of accuracy (Fig. 1D-G).

The infiltration of immune cells is an important feature in the progression of immune-mediated inflammatory diseases (IDD). Numerous immune cells, including macrophages, T cells, and neutrophils, are frequently found in degenerated intervertebral disc tissue. The activation and infiltration of these immune cells could amplify inflammatory reactions, thereby accelerating the degeneration and destruction of intervertebral disc tissue. For instance, macrophages can release various inflammatory cytokines, including TNF-α and IL-1β. These cytokines could then stimulate intervertebral disc cells to increase the production of matrix metalloproteinases, leading to the degradation of the extracellular matrix (Tang et al. 2023; Cao et al. 2021).

**Fig. 2:**
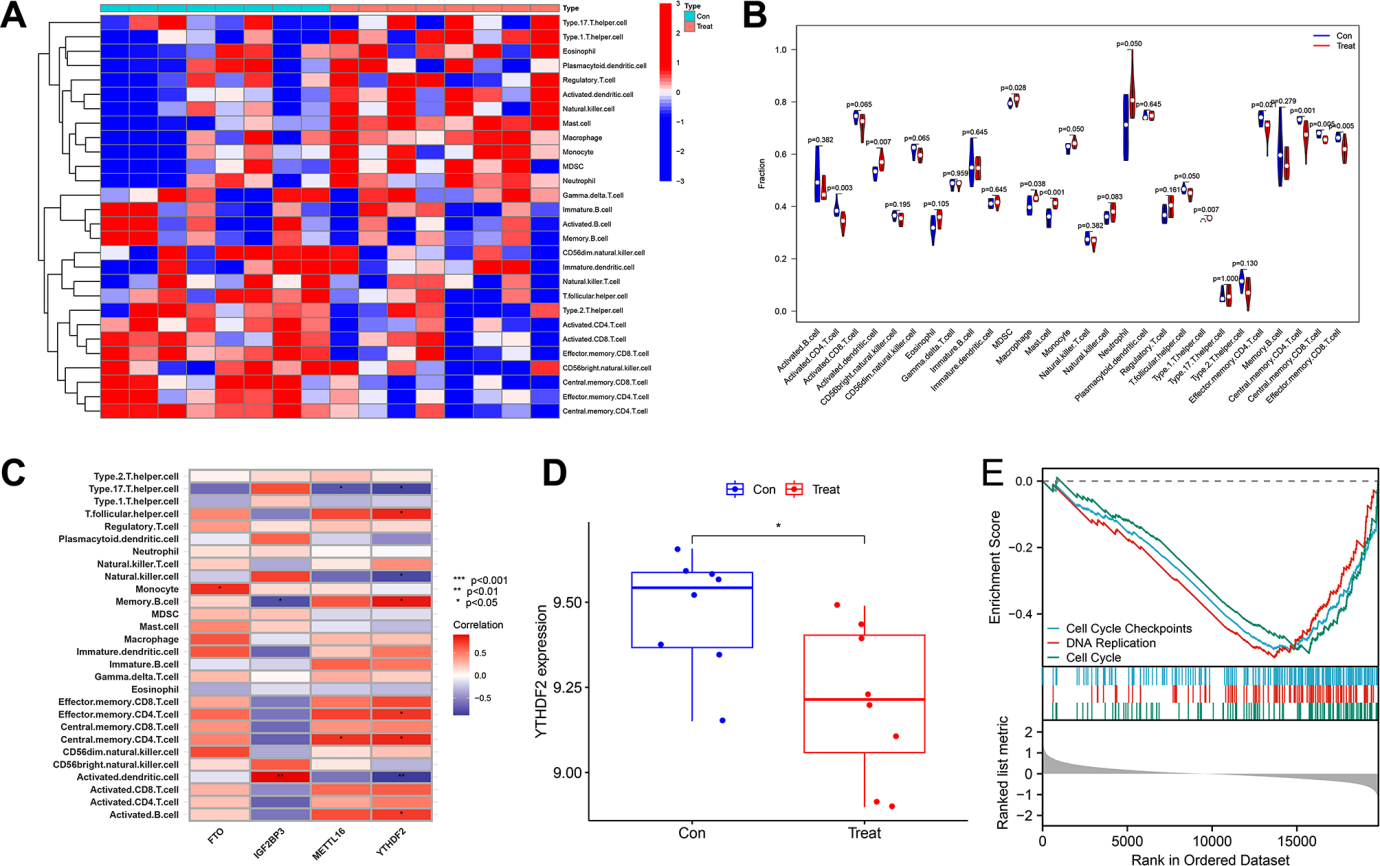
Immune cell infiltration analysis of samples through ssGSEA. Note: **(A)** Heatmap of immune cells in the control group (Control group, normal mice, *n* = 8) and treatment group (Treat group, IDD group, *n* = 8); **(B)** Violin plot analysis of the differences in immune cells between the control group and treatment group; **(C)** Correlation analysis between candidate genes and immune cells; **(D)** Differential expression of YTHDF2 in the control group and treatment group, with a t-test used between the two groups, where * indicates significant differences between the two groups (*P* < 0.05); **(E)** GSEA analysis of differential gene sets between the control group and treatment group

To evaluate the infiltration of immune cells in the samples, we employed ssGSEA (single-sample gene set enrichment analysis) to determine the immune cell content for each sample. The results were then displayed as a heatmap and violin plot (Fig. 2A-B) to illustrate the regulation of immune cells. Moreover, by examining the correlation between these four candidate genes and immune cells, we discovered that YTHDF2 exhibited the strongest association with the number of immune cells (Fig. 2C). Consequently, we designated YTHDF2 as the essential gene for diagnosing IDD. The expression level of YTHDF2 was downregulated in the treatment group when compared to the control group (Fig. 2D).

Gene set enrichment analysis using GSEA revealed that three gene sets, namely Cell Cycle Checkpoints, DNA Replication, and Cell Cycle, were enriched in the control group (Fig. 2E). This observation implies a potential correlation between the cell cycle and intervertebral disc degeneration.

### YTHDF2 modulates oxidative stress-induced aging and proliferation in NP cells: insights from overexpression and knockdown experiments

**Fig. 3:**
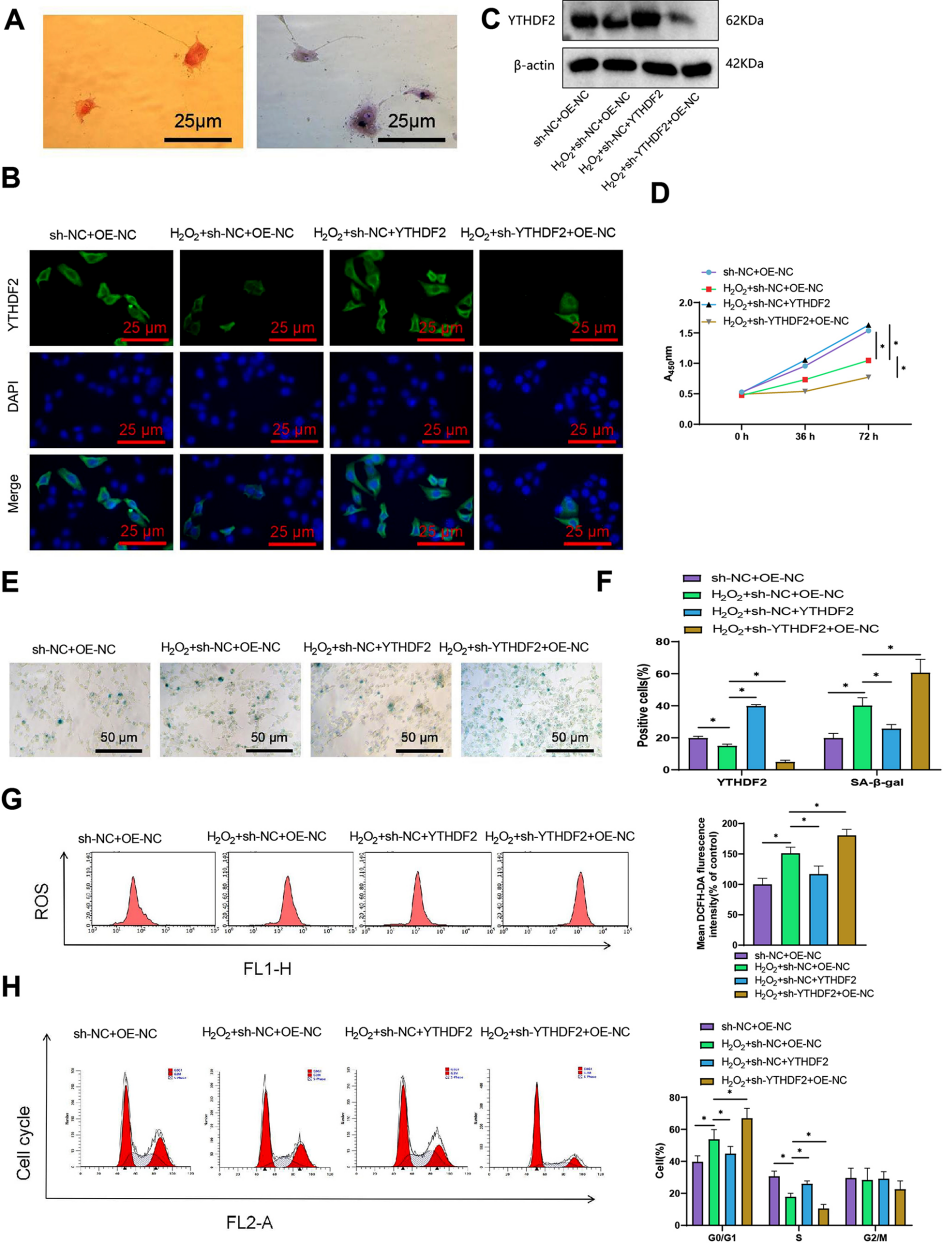
Effects of YTHDF2 on NP cell proliferation, senescence, and reactive oxygen species (ROS) levels. Note: **(A)** Staining identification of NP cells in mice, Safranin O staining shows that the cell nucleus appears orange; Toluidine blue staining shows that the cell nucleus appears blue. The scale bar in the figure is 25 μm; **(B)** Immunofluorescence detection of YTHDF2 expression levels in NP cells under different treatment conditions (sh-NC + OE-NC, H_2_O_2_ + sh-NC + OE-NC, H_2_O_2_ + sh-NC + YTHDF2 and H_2_O_2_ + sh-YTHDF2 + OE-NC). YTHDF2 expression is shown in green fluorescence, and DAPI represents the cell nucleus staining results, with the scale bar labeled as 25 μm (200×); **(C)** Western blot detection of YTHDF2 expression levels under different treatment conditions; **(D)** CCK-8 detection of NP cell viability under different treatment conditions as in A; **(E)** SA-β-gal staining to assess changes in cell aging under different treatment conditions, with the scale bar labeled as 50 μm (200×); **(F)** Statistical comparison of the percentage of positive cells in Figures A and D; **(G)** Flow cytometry to determine ROS levels in NP cells under different treatment conditions with statistical analysis; **(H)** Flow cytometry to determine the cell cycle distribution in NP cells under different treatment conditions; t-test, * indicates a significant difference between the two groups (*P* < 0.05), with *n* = 6 samples in each cell experiment group and the experiment repeated 3 times

To elucidate the role of YTHDF2 in the progression of Intervertebral Disc Degeneration (IDD), NP cells were harvested from 6-week-old mice. After 24 h, primary NP cells began to adhere to the substrate. Observation under a microscope revealed spindle-shaped cells with large, round nuclei and abundant cytoplasm. Safranin O staining depicted the cell nuclei in orange, while toluidine blue staining showed blue nuclei, as depicted in Fig. 3A. These characteristics served to identify the extracted cells as NP cells.

The NP cells were divided into four groups and named as follows: sh-NC + OE-NC group, H_2_O_2_ + sh-NC + OE-NC group, H_2_O_2_ + sh-NC + YTHDF2 group, and H_2_O_2_ + sh-YTHDF2 + OE-NC group. The cells were treated with 100 µM H_2_O_2_ or PBS for 24 h, and different plasmids were transfected with lentivirus to overexpress or knock down YTHDF2 (Du et al. 2022b).

Immunofluorescence and Western blot analyses confirmed that treatment with H_2_O_2_ alone (100 µM) significantly reduced the expression levels of YTHDF2 protein compared to the untreated group (Figure S2A, B, E). Moreover, successful overexpression and knockdown of YTHDF2 were achieved in the H2O2 + sh-NC + YTHDF2 and H2O2 + sh-YTHDF2 + OE-NC groups, respectively (Fig. 3B, C and F). Evaluation of cell senescence and proliferation revealed that treatment with H_2_O_2_ alone profoundly inhibited the vitality of NP cells and promoted cellular senescence compared to the untreated group (Figure S2C-E). Interestingly, the effects of H_2_O_2_ were reversed when YTHDF2 was overexpressed, while they were enhanced upon YTHDF2 knockdown (Fig. 3D-F, Figure S2C-E).

Flow cytometry analysis was performed to measure ROS levels and the cell cycle. The results revealed that treatment with H_2_O_2_ alone significantly increased cellular ROS levels and the proportion of cells in the G0/G1 phase while markedly decreasing the proportion of cells in the S phase compared to the untreated group (Figure S2F-G). Interestingly, these changes were reversed upon overexpression of YTHDF2, and conversely, deepened upon YTHDF2 knockdown (Fig. 3G-H, Figure S2F-G).

In summary, our findings suggest that YTHDF2 may accelerate the cell cycle, promote cell proliferation, and play a role in regulating the aging process by potentially mediating oxidative stress.

### YTHDF2 overexpression attenuates intervertebral disc degeneration in mice: impact on disc structure matrix degradation and inflammatory responses

**Fig. 4:**
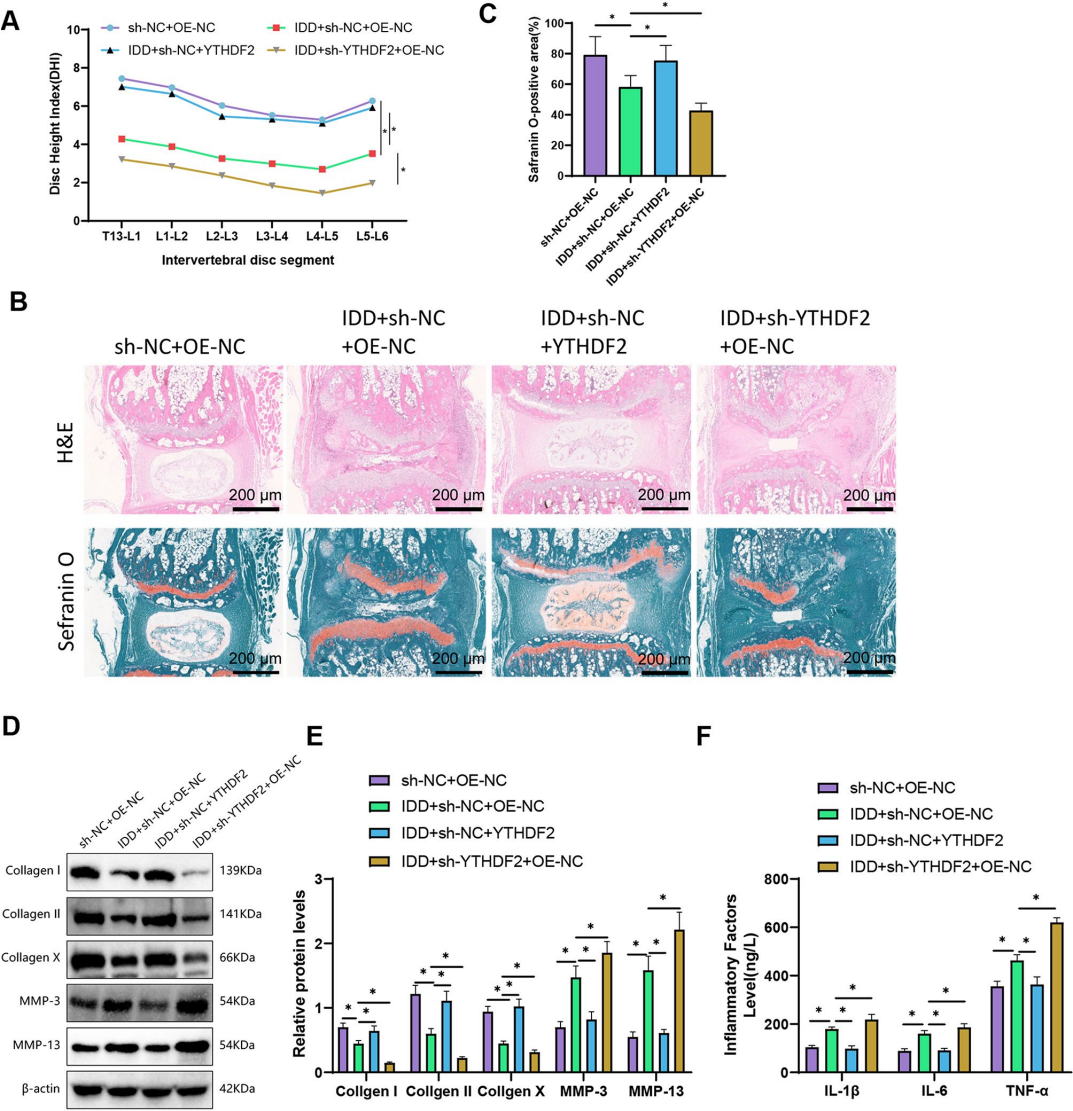
Regulatory role of YTHDF2 in mouse IDD. Note: **(A)** Representative X-ray images under different treatment conditions of each group of mice (sh-NC + OE-NC, IDD + sh-NC + OE-NC, IDD + sh-NC + YTHDF2, and IDD + sh-YTHDF2 + OE-NC) were used to calculate the disc height index (DHI) of mouse lumbar intervertebral discs; **(B)** H&E staining and Safranin O staining images of lumbar intervertebral discs for different groups, where orange indicates NP cells and collagen, blue represents fibers, with a scale bar of 200 μm (200×); **(C)** Size of positive areas for Safranin O staining in different groups; **(D)** Western blot analysis of protein expression levels in different groups; **(E)** Graphical representation of protein expression levels in different groups; **(F)** ELISA analysis of inflammatory factors IL-1β, IL-6, and TNF-α expression levels in disc tissue. Each group consisted of *n* = 6 mice; t-test, * indicates significant difference between two groups (*P* < 0.05), with the experiment repeated 3 times

To further evaluate the preventive role of YTHDF2 in vivo in intervertebral disc degeneration (IDD), we established a mouse IDD model using tail suspension (TS) and achieved overexpression or knockdown of YTHDF2 by intravenous injection of lentivirus. The mice were divided into four groups: sh-NC + OE-NC, IDD + sh-NC + OE-NC, IDD + sh-NC + YTHDF2, and IDD + sh-YTHDF2 + OE-NC. Four weeks after tail suspension (TS), we observed congestion injury in the surrounding muscles of mice with intervertebral disc degeneration (IDD). We then performed X-ray imaging to calculate the subsequent Disc Height Index (DHI) (Fig. 4A).

The lumbar intervertebral disc was assessed using H&E staining and Safranin O staining. The results revealed a decrease in the Safranin O positive area in mice from the IDD + sh-NC + OE-NC group compared to the control group. However, the overexpression of YTHDF2 reversed this change, whereas knockdown had the opposite effect. The findings from H&E staining were consistent with these observations (Fig. 4B-C). The DHI of the IDD + sh-NC + OE-NC group mice was reduced compared to the control group mice, as determined by the calculation. However, overexpression of YTHDF2 was found to alleviate this situation, while knockdown had the opposite effect. (Fig. 4A).

Matrix metalloproteinases (MMPs) can degrade all types of extracellular matrix proteins, reducing the levels of aggregated proteins and collagen within tissues. During intervertebral disc degeneration, collagen proteins’ structure and regenerative function are compromised (Li et al. 2022a). Hence, we evaluated the protein expression levels of MMP3, MMP13, Collagen I, Collagen II, and Collagen X. The results demonstrated an increase in the protein levels of these MMPs in the IDD model mice, whereas the protein levels of the three collagen above types decreased significantly. The overexpression of YTHDF2 partially reversed these changes, whereas knockdown had the opposite effect (Fig. 4D-E).

During immune-mediated inflammatory diseases (IDD), inflammatory reactions frequently worsen. We utilized the Elisa method to measure the levels of inflammatory factors in the NP tissue of mice. Our findings indicate that IDD augmented the expression of inflammatory factors IL-1β, IL-6, and TNF-α in mice. Overexpression of YTHDF2 leads to a reduction in the expression of these inflammatory factors, whereas knockdown has the opposite effect (Fig. 4F).

In summary, the overexpression of YTHDF2 mitigates the severity of intervertebral disc degeneration (IDD) in mice.

### YTHDF2 counteracts oxidative stress and promotes cell proliferation in nucleus pulposus cells: insights from protein expression and cellular mechanisms

**Fig. 5:**
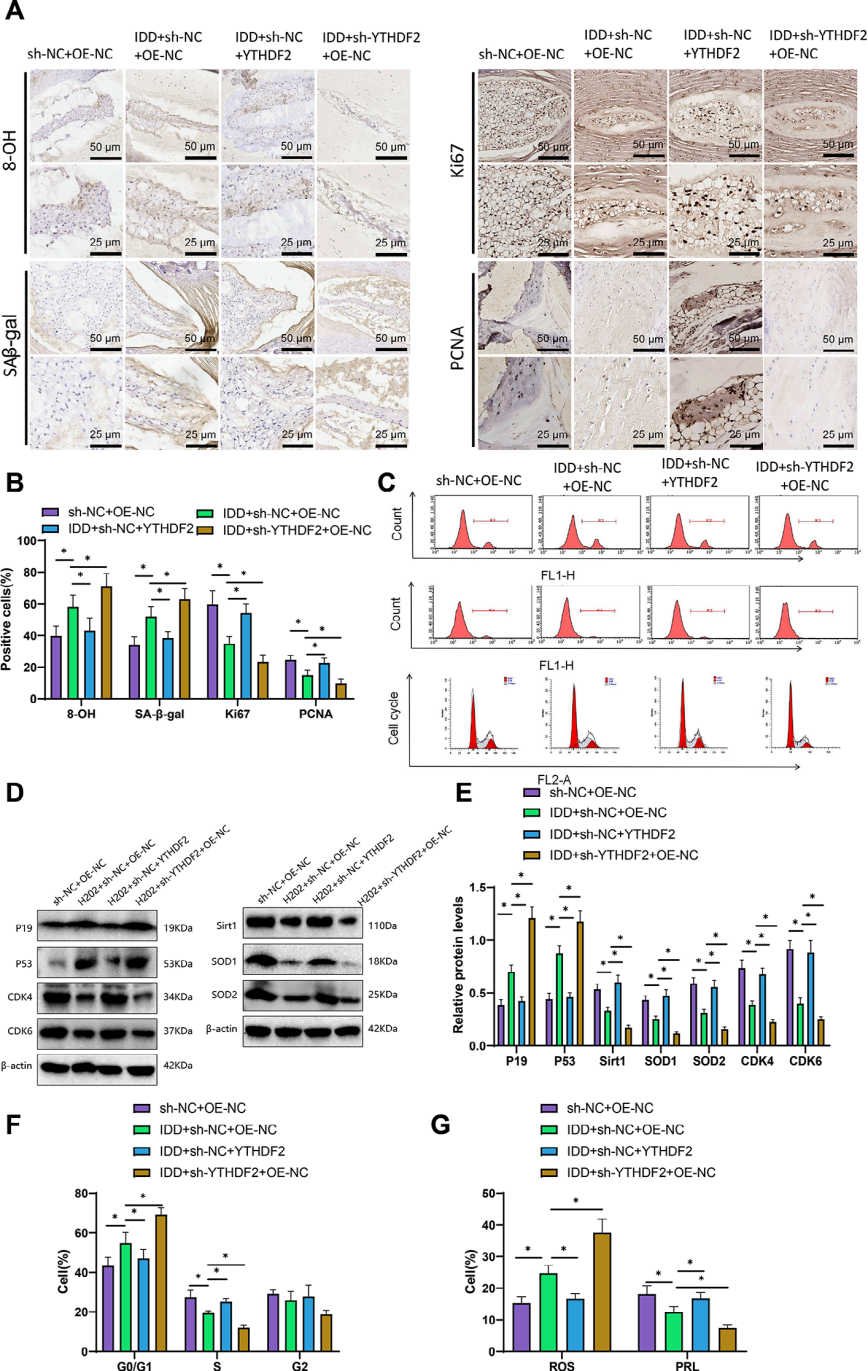
Effects of YTHDF2 on antioxidant capacity and NP cell proliferation in mice. Note: **(A)** Immunohistochemical staining was performed on intervertebral disc slices from different groups (sh-NC + OE-NC, IDD + sh-NC + OE-NC, IDD + sh-NC + YTHDF2, and IDD + sh-YTHDF2 + OE-NC), respectively labeled with 8-hydroxy-2’-deoxyguanosine (8-OHdG), senescence-related beta-galactosidase (SA-β-gal), Ki67, and proliferating cell nuclear antigen (PCNA), shown as 50 μm (200×, top) or 50 μm (200×, bottom); **(B)** Statistical graphs of immunohistochemical staining in different groups; **(C)** Measurement of reactive oxygen species (ROS) levels, cell proliferation (PRL), and cell cycle distribution in freshly collected mouse NP cells under different treatments by flow cytometry; **(D)** Detection of different protein expression levels by Western blot under different treatment conditions; **(E)** Statistical graphs of protein expression levels under different treatment conditions; **(F)** Flow cytometry analysis of cell cycle distribution under different treatment conditions; **(G)** Quantitative statistical graphs of ROS levels and PRL levels under different treatment conditions; the number of mice in each group is *n* = 6; t-test, * denotes significant difference between two groups (*P* < 0.05). The experiment was repeated three times

In our in vitro experiments, we discovered that YTHDF2 has a regulatory role in nucleus pulposus (NP) cells. To further investigate the potential regulatory mechanisms of YTHDF2 on NP cells, we conducted immunohistochemical staining on intervertebral disc tissues from mice. The results suggest that IDD increases the proportion of 8-OH and SA-β-gal positive cells while simultaneously inhibiting the proportion of Ki67 and PCNA positive cells. It indicates that IDD promotes cellular aging and oxidation while inhibiting cell proliferation. Overexpression of YTHDF2 has the potential to reverse this alteration (Fig. 5A-B).

Using flow cytometry, we detected increased levels of reactive oxygen species (ROS), reduced cell proliferation (PRL), and inhibited cell cycle progression in mice with IDD compared to the control group. IDD increased ROS levels while reducing the number of cells transitioning from the G0/G1 phase to the S phase and impeding cell proliferation. Overexpression of YTHDF2 could effectively reverse this change, whereas knockdown of YTHDF2 produces the opposite effect (Fig. 5C, F and G).

The protein expression of CDK4, CDK6, P19, P53, and antioxidant enzymes Sirt1, SOD1, and SOD2 was analyzed using Western blot. The results demonstrated that IDD markedly suppressed the protein expression of CDK4, CDK6, Sirt1, SOD1, and SOD2 while concurrently upregulating the P19 and P53 protein expression levels. The overexpression of YTHDF2 reversed this change, while the knockdown of YTHDF2 had the opposite effect (Fig. 5D-E). These results are consistent with previous findings.

In summary, YTHDF2 demonstrates antioxidant effects in mice and stimulates NP cell proliferation.

### O-GlcNAcylation of YTHDF2 at Ser263 modulates its function in NP cells: insights from ogt interaction and stress responses

In previous studies, we have identified the roles of YTHDF2 in regulating oxidative stress and the cell cycle. We conducted the following experimental investigations to examine the impact of O-GlcNAc glycosylation modification on the activity and function of YTHDF2 in cells and tissues, as well as its potential regulatory mechanisms in IDD.

**Fig. 6:**
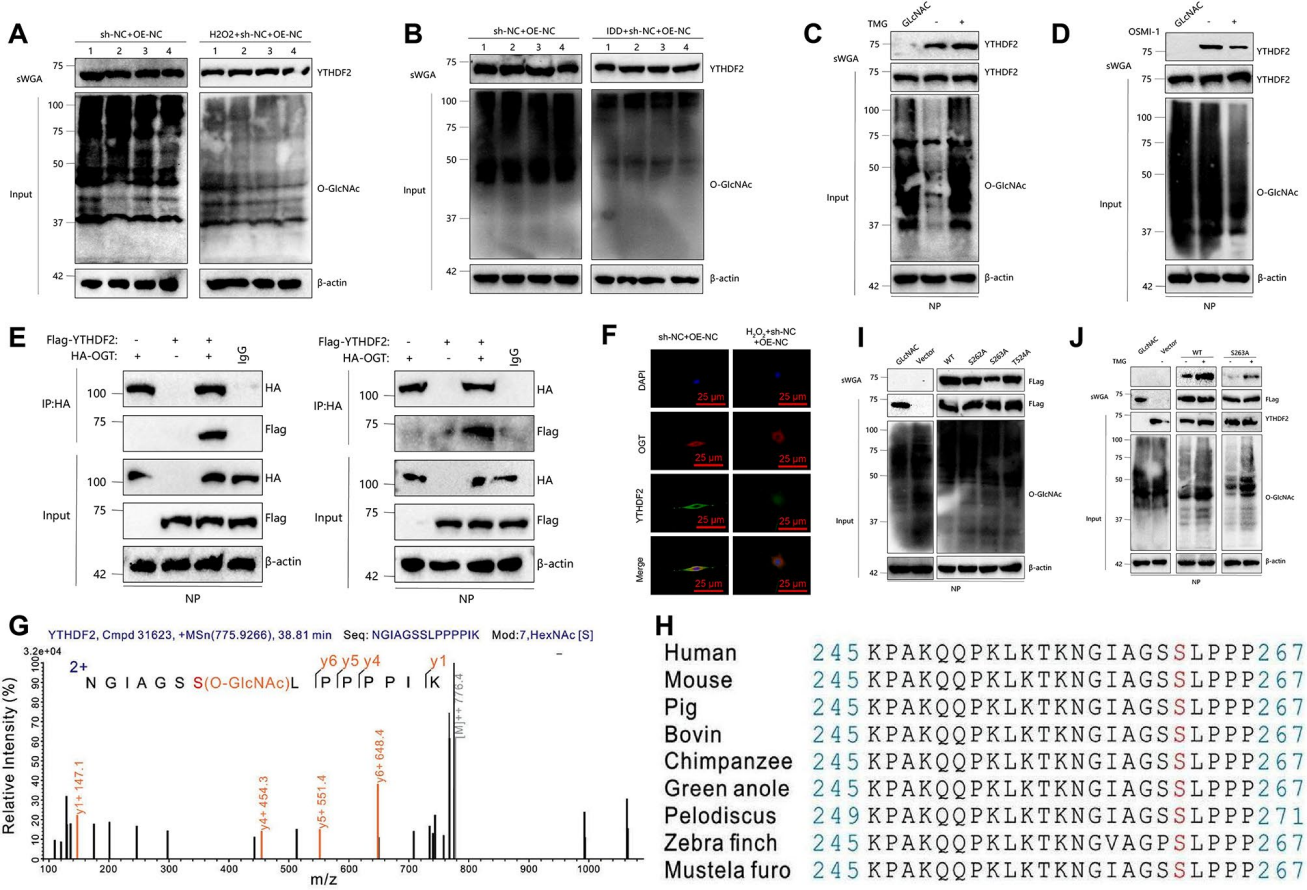
Regulation of O-GlcNAcylation of YTHDF2 in H_2_O_2_-induced NP cells and IDD mice. Note: **(A-B)** O-GlcNAcylation of YTHDF2 and total O-GlcNAcylation in H_2_O_2_-induced NP cells and IDD mice were determined by sWGA pull-down assay. **(C-D)** Changes in O-GlcNAcylation were detected in NP cells treated with 25 µM TMG or 20 µM OSMI-1 for 12 h. **(E)** Co-IP confirmed the interaction between YTHDF2 and OGT. **(F)** Co-localization of YTHDF2 and OGT, scale bar represents 25 μm (400×). **(G)** LC-MS analysis identified Ser263 as the O-GlcNAc site on YTHDF2. **(H)** Cross-species sequence alignment of YTHDF2. **(I)** sWGA assay was performed in NP cells transfected with plasmids encoding Flag-tagged YTHDF2 (WT, S262A, S263A, or T524A) using an anti-Flag antibody. **(J)** NP cells were transfected with Flag-tagged YTHDF2 (WT or S263A) and subjected to sWGA assay. Each experiment was repeated 3 times, *n* = 6 mice per group. * indicates differences between groups (*P* < 0.05)

In this study, we initially measured the levels of YTHDF2 O-GlcNAc and total O-GlcNAc in NP cells and mice using sWGA experiments. We observed that both H_2_O_2_ induction and IDD resulted in reduced levels of YTHDF2 O-GlcNAc and total O-GlcNAc (Fig. 6A-B). Subsequently, NP cells were treated separately with the OGT inhibitor OSMI-1 and the OGA inhibitor TMG. We discovered that TMG increased the overall levels of O-GlcNAc and the O-GlcNAc levels of YTHDF2. In contrast, treatment with OSMI-1 had the opposite effect (Fig. 6C-D). It suggests that the overall O-GlcNAc level within the cellular environment controls the O-GlcNAc level of YTHDF2. OGT is the sole enzyme responsible for promoting protein O-GlcNAc modification.

We initially conducted Co-IP experiments to provide evidence for the O-GlcNAcylation of YTHDF2 being mediated by OGT. These experiments revealed an interaction between OGT and YTHDF2 (Fig. 6E). Additionally, a co-localization trend of OGT and YTHDF2 in cells was observed through confocal microscopy experiments. Treatment with H_2_O_2_ reduces the interaction between YTHDF2 and OGT, providing additional evidence of their potential involvement in the same cellular pathway (Fig. 6F).

LC-MS analysis was conducted on the immunoprecipitates of YTHDF2 with a Flag tag. The analysis revealed that Ser263 (S263) is the primary O-GlcNAc site of YTHDF2, as shown in Fig. 6G. Additionally, Fig. 6H demonstrates the conservation of this site in different species.

Moreover, we generated vectors containing YTHDF2 sequences with various point mutations (WT, S262A, S263A, T524A) and transfected these vectors into NP cells. Our findings indicate that the substitution of Ser362 with Ala (S263A) reduced the O-GlcNAc level of YTHDF2 (Fig. 6I), and TMG treatment no longer resulted in an increase in the O-GlcNAc level of YTHDF2 (Fig. 6J).

The experiments above have demonstrated that both H_2_O_2_ induction and IDD enhance the O-GlcNAcylation level of YTHDF2 through modification at the S263 site.

### O-GlcNAcylation enhances YTHDF2 Stability by inhibiting ubiquitination without affecting subcellular localization

O-GlcNAc glycosylation regulates target proteins’ localization, stability, and protein-protein interactions, thereby participating in a wide range of cellular processes and signal transduction pathways. To explore the influence of O-GlcNAc on YTHDF2, we initially analyzed the native YTHDF2 in NP cells treated with the O-GlcNAcase (OGA) inhibitor TMG. The results demonstrated that TMG reversed the decrease in YTHDF2 protein expression caused by H_2_O_2_ (Figure S3A). Moreover, the protein level of YTHDF2 was downregulated following OGT knockdown (Figure S3B). These findings suggest that the downregulation of YTHDF2 induced by H_2_O_2_ primarily depends on OGT-mediated O-GlcNAcylation. Protein degradation pathways in eukaryotic cells primarily comprise the ubiquitin-proteasome and lysosomal pathways.

Our findings demonstrate that the proteasome inhibitor MG132, when administered alone, effectively counteracts the downregulation of YTHDF2 caused by OSMI-1 treatment (Figure S3C). Thus, we propose that O-GlcNAc potentially regulates the expression of YTHDF2 via the ubiquitin-proteasome pathway.

Afterward, we transfected Flag-tagged YTHDF2 into NP cells treated with shOGT or shOGA for subsequent processing. The analysis of chloramphenicol (CHX) tracking revealed that silencing OGT resulted in accelerated degradation of the YTHDF2 protein, whereas silencing OGA led to a slowdown in its degradation rate (Figure S3D).

**Fig. 7:**
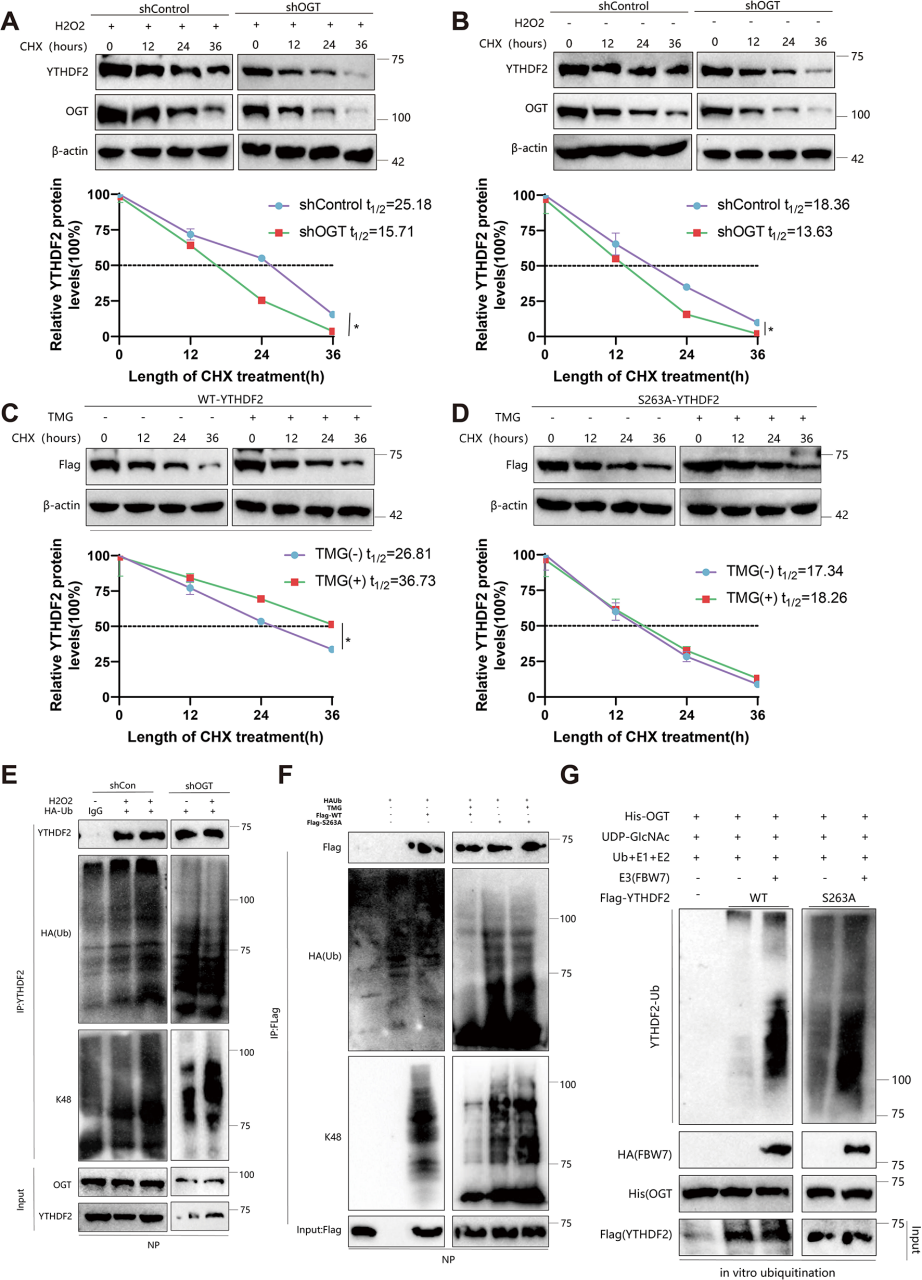
O-GlcNAcylation stabilizes YTHDF2 by inhibiting its ubiquitination. Note: **(A-B)** NP cells were transfected with OGT shRNA lentivirus and treated with 2 µM cycloheximide (CHX) for the indicated periods. The half-life of YTHDF2 was determined by immunoblotting. **(C-D)** The half-life of Flag-YTHDF2 and quantitative analysis were performed in NP cells treated or untreated with 25 µM TMG. **(E)** In NP cells transfected with OGT shRNA lentiviral vector, in vivo ubiquitination of YTHDF2 was assessed under HA-tagged ubiquitin (HA-Ub) conditions. **(F)** NP cells transfected with Flag-YTHDF2 (WT or S263A) were treated with 25 µM TMG. Before harvest, cells were treated with 10 µM MG132 to prevent degradation. Immunoprecipitation of YTHDF2 was performed using YTHDF2 or Flag antibody. **(G)** in vitro ubiquitination of YTHDF2 was performed using purified Flag-tagged WT or S263A YTHDF2, His-OGT, SCFFBW7 E3 complex, E1, E2, Ub, and UDP-GlcNAc. The reaction products were subjected to immunoblotting, and YTHDF2 ubiquitination was detected using the YTHDF2 antibody. Each experiment was repeated 3 times. A two-way analysis of variance was conducted to compare the expression levels at different time points between the two groups. * indicates differences between groups (*P* < 0.05)

Furthermore, the endogenous YTHDF2 protein is more susceptible to degradation following H_2_O_2_ treatment. However, the knockdown of OGT reduces the half-life of YTHDF2, regardless of whether it is treated with H_2_O_2_ or not (Fig. 7A-B).

Moreover, the S263A mutation decreased the half-life of YTHDF2 when compared to YTHDF2-WT. On the other hand, TMG treatment only extended the half-life of YTHDF2-WT, having little impact on the S263A variant (Fig. 7C-D). These findings indicate that treatment with H_2_O_2_ promotes the degradation of the YTHDF2 protein via downregulation of O-GlcNAc glycosylation.

We further investigated the influence of O-GlcNAc on the ubiquitination of YTHDF2. The ubiquitination of YTHDF2, in terms of total protein and K48-linked ubiquitination, is increased by H_2_O_2_ treatment. Additionally, the ubiquitination level is further elevated by transfection with shOGT, as shown in Fig. 7E. Moreover, the S263A mutation in YTHDF2-WT substantially enhanced YTHDF2 ubiquitination, whereas TMG solely impacted WT ubiquitination, displaying a negligible effect on S263A mutation (Fig. 7F). FBW7 has been identified as a mediator of YTHDF2 protein degradation (Xu et al. 2021).

To further validate the impact of O-GlcNAc on YTHDF2 ubiquitination, we conducted an in vitro ubiquitination assay using the E3-ubiquitin ligase F-box and WD repeat domain protein 7 (FBW7) (Fig. 7G). The S263A mutation markedly enhances the ubiquitination level of YTHDF2 in comparison to YTHDF2-WT, facilitated by FBW7. These findings suggest that O-GlcNAcylation of Ser263 could enhance the stability of YTHDF2 by inhibiting its ubiquitination process.

Furthermore, we also evaluated the potential influence of O-GlcNAcylation on the subcellular distribution of YTHDF2. NP cells’ nuclear/cytoplasmic proteins were extracted using a reagent kit. The results of the nuclear/cytoplasmic separation experiment and immunofluorescence experiment demonstrated that TMG or OSMI-1 primarily affected the expression of YTHDF2 while having minimal impact on its localization (Figure S3E-F). No difference in subcellular localization between the WT and S263A variants was observed, as shown in Figure S3G-H.

The results suggest that O-GlcNAc modification increases the stability of the YTHDF2 protein while having minimal impact on its subcellular localization.

### YTHDF2’s S263A mutation exacerbates intervertebral disc degeneration by inhibiting NP cell proliferation and affecting cell cycle progression

Previous studies have demonstrated the regulatory role of YTHDF2 in NP cell proliferation and mouse IDD deterioration. Furthermore, we discovered that the O-GlcNAcylation of YTHDF2 could augment its protein stability. Therefore, we conducted experiments to investigate the involvement of YTHDF2 O-GlcNAcylation in regulating IDD. The experiment was divided into four groups: sh-control, shYTHDF2, shYTHDF2 + WT, and shYTHDF2 + S263A.

**Fig. 8:**
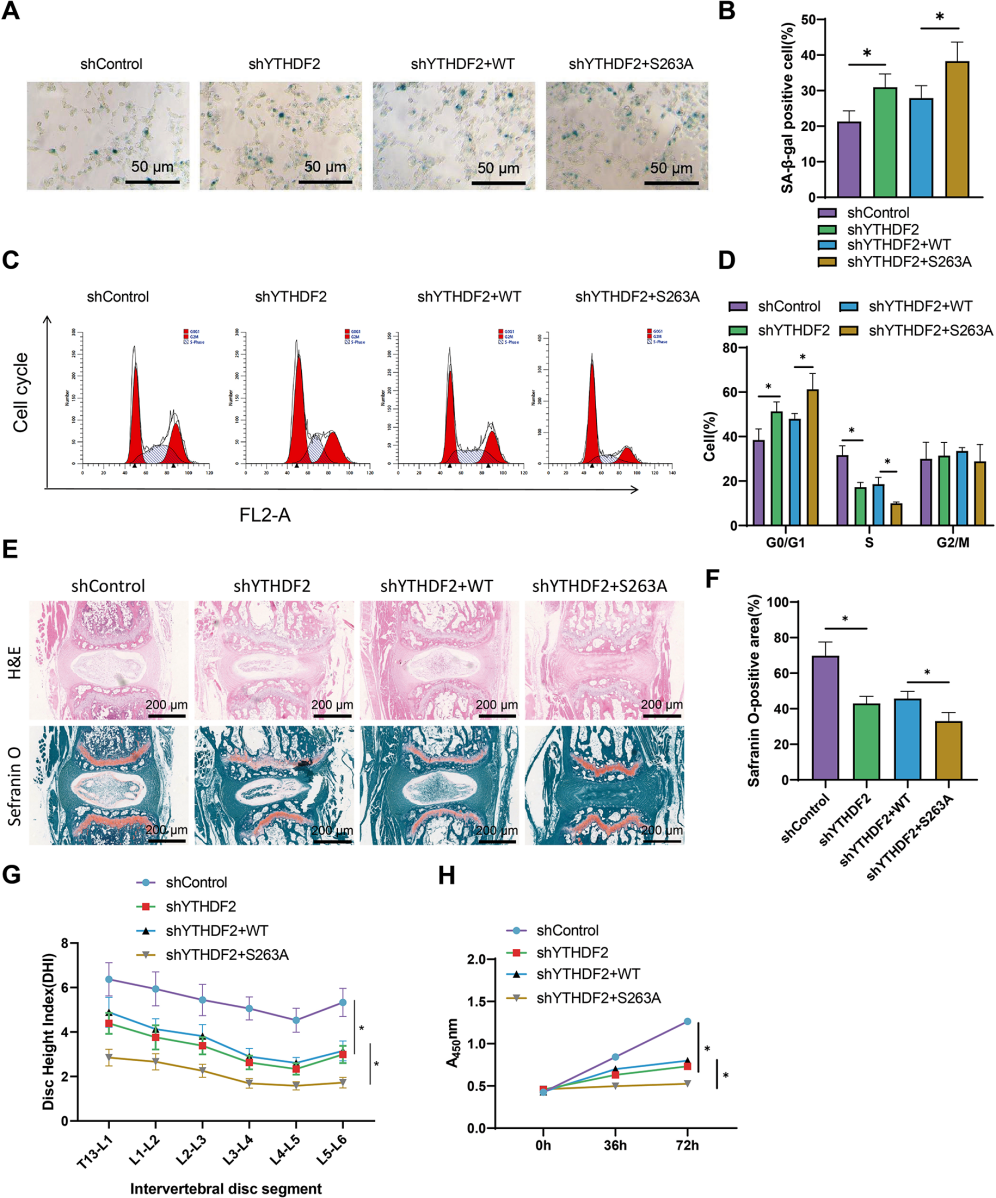
Regulation of NP cell proliferation and IDD mice by O-GlcNAcylation of YTHDF2. Note: **(A-B)** NP cells were induced for endogenous YTHDF2 knockdown by transfection with YTHDF2 shRNA lentiviral vector, followed by infection with plasmid vectors expressing Flag-YTHDF2 (WT or S263A). The senescence of NP cells was assessed by SA-β-gal staining and statistical analysis (Scale bar = 50 μm). **(C-D)** Flow cytometry was performed to determine the cell cycle distribution of NP cells, and statistical analysis was conducted. **(E-F)** Different lentiviruses were injected into IDD mice via the tail vein, and intervertebral discs were subjected to H&E and Safranin O staining. Corresponding images and statistical analysis were generated. Orange represents NP cells and collagen, and blue represents fibers (Scale bar = 50 μm). **(G)** Disc height index (DHI) of each group of mice. **(H)** NP cell proliferation capacity was assessed using CCK-8 assay. Each group of mice consisted of *n* = 6, and each experimental group was repeated 3 times. Expression levels at different time points were assessed using a two-way analysis of variance, and comparisons involving three groups were analyzed using a one-way analysis of variance. * indicates differences between groups (*P* < 0.05), *n* = 6 for NP cell experiments

In line with prior research, the number of senescent cells shows an increase upon depletion of YTHDF2. Co-transfecting cells with the YTHDF2 S263A plasmid further increased the number of senescent cells (Fig. 8A-B). Flow cytometry analysis demonstrated that the S263A mutation in YTHDF2 caused cell arrest in the G0/G1 phase, accompanied by a substantial reduction in the number of cells in the S phase, impeding cell cycle progression (Fig. 8C-D). Furthermore, the results of the CCK-8 experiment confirmed these findings, demonstrating that the S263A mutation inhibited the proliferation capacity of NP cells (Fig. 8H).

Lentiviruses were injected into the IDD mice through the caudal vein, followed by H&E staining and Safranin O staining of the intervertebral discs. Subsequently, the DHI index of the mice was computed. The results demonstrated that the S263A mutation further decreased the proportion of Safranin O-positive areas and the DHI index in mice with IDD (Fig. 8E-G).

In conclusion, the S263A mutation in YTHDF2 inhibits the proliferation of nucleus pulposus (NP) cells and further exacerbates intervertebral disc degeneration (IDD) in mice.

### YTHDF2 O-GlcNAc modification enhances CCNE1 mRNA stability and influences cell cycle progression via direct interaction

YTHDF2 regulates CCNE1 mRNA stability through O-GlcNAc modification. YTHDF2 is a key reader of m6A modifications, enabling it to recognize dynamic m6A modifications and regulate the stability and translation status of methylated mRNA. W poprzedniej sekcji analizy bioinformatycznej uzyskano 3441 DEG (genów różnicowo ekspresyjnych) z komórek NP myszy z IDD i grupy kontrolnej przy użyciu sekwencjonowania wysokoprzepustowego oraz analizy GSEA. Ponadto zidentyfikowano również trzy zestawy genów, mianowicie Kontrolki cyklu komórkowego, Replikację DNA oraz Cykl komórkowy.

**Fig. 9:**
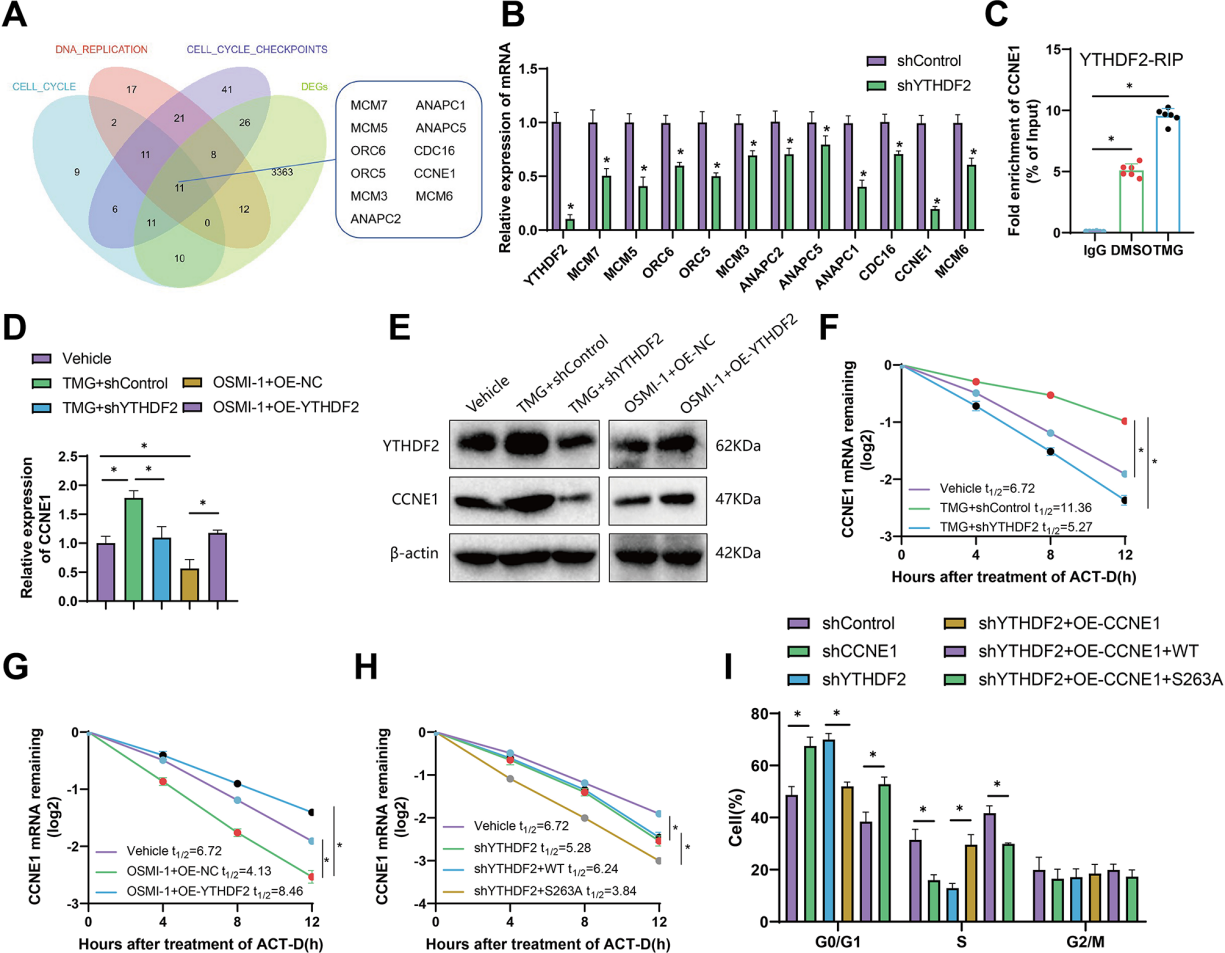
Impact of YTHDF2 O-GlcNAcylation on CCNE1 transcript. Note: **(A)** Venn diagram was used to screen potential downstream target genes of YTHDF2. **(B)** mRNA changes of potential target genes were analyzed in NP cells transfected with YTHDF2 shRNA lentivirus. **(C)** YTHDF2-RIP-qPCR experiment showed an association between CCNE1 transcript and YTHDF2 in NP cells. **(D-E)** Changes in CCNE1 mRNA and protein levels after YTHDF2 knockdown or overexpression and treatment with 25 µM TMG or 20 µM OSMI-1. **(F-H)** CCNE1 mRNA half-life was determined in NP cells treated with control or YTHDF2 shRNA, followed by either 25 µM TMG treatment **(F)** or overexpression of YTHDF2 with 20 µM OSMI-1 treatment **(G)**. CCNE1 mRNA half-life was also determined in NP cells transfected with Flag-tagged YTHDF2 (WT or S263A), using transcription inhibition by actinomycin D (5 µg/mL) **(H)**. **(I)** Flow cytometry analysis was performed in NP cells transfected with different lentiviral vectors. Expressions at different time points were analyzed using a two-way analysis of variance, while pairwise group data were assessed with independent sample t-tests, and comparisons involving three groups were analyzed using a one-way analysis of variance.* indicates differences between groups (*P* < 0.05), *n* = 6 for NP cell experiments, and each experimental group was repeated 3 times

To identify the potential targets regulated by YTHDF2, the Venn diagram was used to intersect the differentially expressed genes (DEGs) and the genes included in these three gene sets. This analysis identified 11 potential targets of YTHDF2 (Fig. 9A). Knockdown of YTHDF2 led to a decrease in the mRNA level of CCNE1 (Fig. 9B).

To ascertain whether CCNE1 is a direct target of YTHDF2, we conducted RNA immunoprecipitation followed by quantitative PCR (RIP-qPCR) in NP cells using anti-YTHDF2 antibody (Fig. 9C). The findings confirmed a direct interaction between YTHDF2 protein and CCNE1 mRNA, potentiated following TMG treatment.

The mechanism through which YTHDF2 regulates CCNE1 expression via O-GlcNAc modification was investigated by treating NP cells with the OGA inhibitor TMG and subsequently administering YTHDF2 shRNA. The findings demonstrated that the treatment with TMG led to an increase in the levels of CCNE1 mRNA. Conversely, the effects of TMG were countered by the knockdown of YTHDF2. In contrast, the levels of CCNE1 mRNA were decreased by treatment with OSMI-1, an OGT inhibitor. This decrease was found to be reversible through the overexpression of YTHDF2. Similar results were observed in the protein expression levels of CCNE1 (Fig. 9D-E).

We analyzed mRNA stability in NP cells treated with ACT-D to investigate the potential regulatory role of YTHDF2 on CCNE1 mRNA stability. Following TMG treatment, the half-life of CCNE1 was prolonged but shortened due to shYTHDF2 treatment (Fig. 9F). In contrast, the treatment with OSMI-1 reduced the half-life of CCNE1 mRNA, while its overexpression of YTHDF2 prolonged the half-life (Fig. 9G).

Moreover, introducing the S263A mutation in YTHDF2 via transfection with shYTHDF2 reduces the half-life of CCNE1 mRNA (Fig. 9H). Flow cytometry analysis revealed that downregulation of CCNE1 led to cell cycle arrest in the G0/G1 phase, whereas upregulation promoted cell transition to the S phase. However, this transition was inhibited by the S263A mutation (Fig. 9I).

Combining the experiments above, it could be inferred that YTHDF2 O-GlcNAc extends the half-life of CCNE1 mRNA and enhances its stability.

### O-GlcNAcylation of YTHDF2 in IDD mice enhances protein stability and upregulates CCNE1 expression alleviating IDD severity

**Fig. 10:**
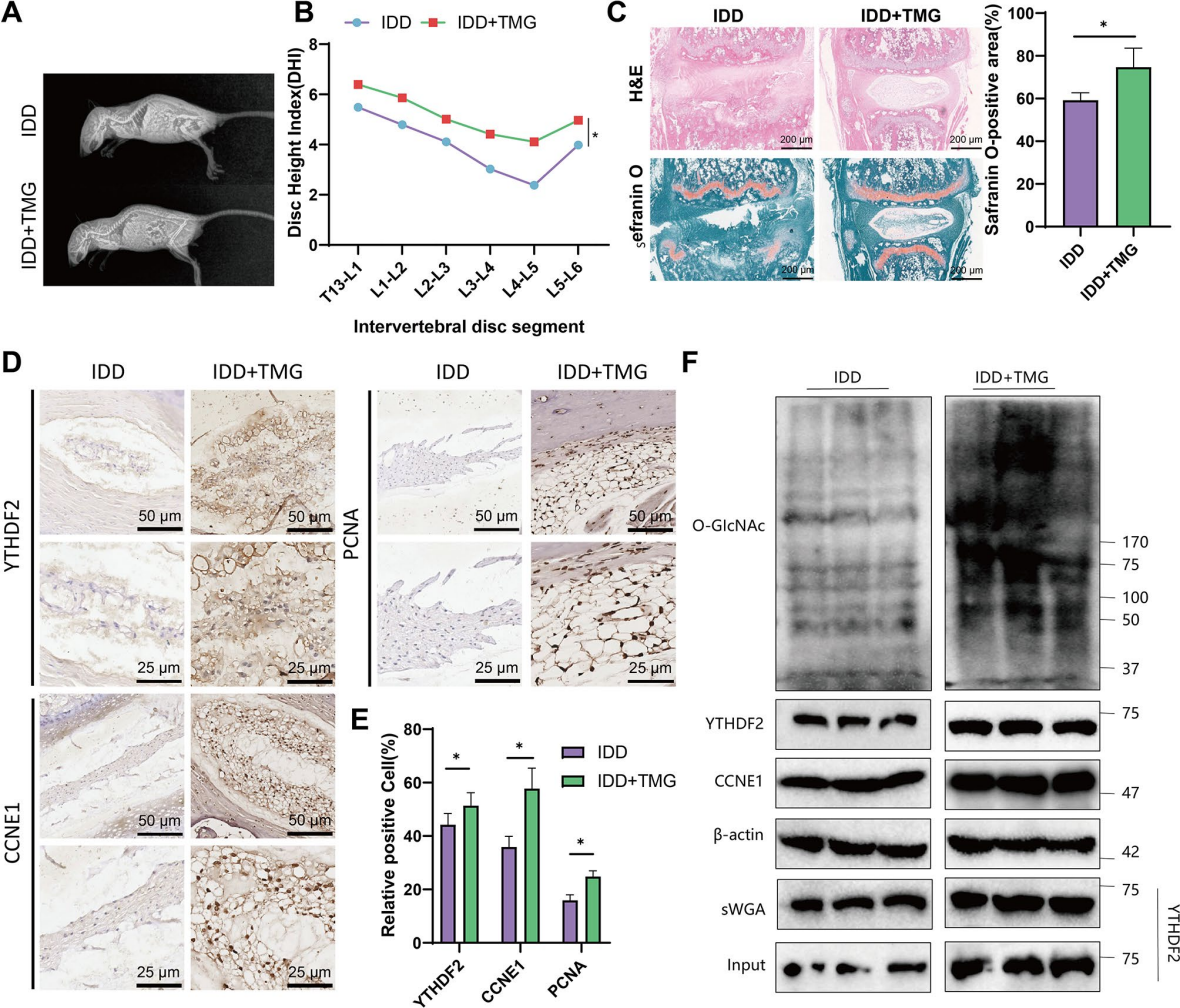
Attenuation of IDD Severity in Mice by TMG. Note: **(A)** Representative X-ray images of IDD mice and IDD + TMG mice; **(B)** Intervertebral disc height index (DHI) of mice in each group; **(A)** H&E and Safranin O staining of lumbar intervertebral discs with corresponding statistical graphs, where orange represents NP cells and collagen, blue represent fibers, and the scale bar in the images is 200 μm (200×); **(D-E)** Representative images and statistical graphs of immunohistochemical staining of lumbar intervertebral disc slices, with the scale bar at 50 μm (200×, top row) or 25 μm (200×, bottom row); **(F)** Protein expression levels in IDD mice and IDD + TMG mice detected by Western blot; *n* = 6 mice per group in the experiment. Expression levels at different time points were analyzed using repeated measures analysis of variance, with independent sample t-tests conducted for comparisons between two groups. * indicates differences between groups (*P* < 0.05)

To confirm the conclusion above, we administered the targeted inhibitor of OGA, TMG, through intraperitoneal injection in IDD mice. X-ray imaging allows for the calculation of the DHI in mice. It is evident from Fig. 10A-B that TMG increases the average DHI index in IDD mice.

H&E staining and Safranin O staining were conducted on slices of lumbar intervertebral discs (IDD) from mice. TMG notably enlarged the Safranin O-positive area, as observed in Fig. 10C. Immunohistochemistry and Western blot analyses demonstrate an upregulation of total O-GlcNAc and YTHDF2 O-GlcNAc levels following TMG treatment. Moreover, the protein levels of YTHDF2, CCNE1, and PCNA were all found to be increased (Fig. 10D-F).

The experimental results above suggest that O-GlcNAcylation of YTHDF2 enhances the stability of the YTHDF2 protein and increases the expression of the CCNE1 protein. As a result, this alleviates the severity of lesions in IDD mice. These findings are consistent with the conclusions drawn from in vitro experiments.

## Discussion

Previous studies have mainly implicated YTHDF2 in regulating RNA metabolism and stability (Yu et al. 2021). Our research has demonstrated that YTHDF2 plays a crucial role in the progression of intervertebral disc degeneration (IDD) (Li et al., 2022b). Bioinformatics analysis reveals a difference in the expression of YTHDF2 in NP cells between IDD mice and normal mice, suggesting its potential as an important IDD diagnostic gene (Ma et al. 2023). It offers a novel viewpoint on the involvement of YTHDF2 in the initiation and advancement of diseases compared to prior knowledge (Qin et al., 2023).

GlcNAc glycosylation modification is an essential post-translational modification process involving the attachment of N-acetylglucosamine (GlcNAc) monosaccharides to serine or threonine residues of proteins via β-O-glycosidic bonds. This dynamic balance of O-GlcNAc modification is regulated by O-GlcNAc transferase (OGT) and O-GlcNAcase (OGA). Such modification plays a pivotal role in various physiological and pathological processes, including cellular stress responses, signal transduction, protein stability, transcriptional regulation, and cell cycle progression (Saha et al. 2021). While early studies have validated the crucial role of protein O-GlcNAc modification in several cellular processes, the specific function of this modification in YTHDF2 has yet to be fully understood (Lin et al. 2022). Our study observed a decrease in the O-GlcNAc modification of YTHDF2 in nucleus pulposus (NP) cells upon H_2_O_2_ induction. This novel finding offers crucial insights for further investigating the role of O-GlcNAc modification in cellular function and disease.

Cell cycle regulation is essential for controlling cell proliferation, differentiation, and apoptosis (Liebl and Hofmann 2021). Alterations in the cell cycle are closely associated with the progression of intervertebral disc degeneration in the pathogenesis of IDD (Xin et al. 2022). Compared to previous studies, our findings indicate that YTHDF2 enhances CCNE1 mRNA stability by promoting O-GlcNAc modification, thereby regulating the cell cycle. This finding offers a novel mechanistic insight into IDD formation.

Oxidative stress plays a crucial role in developing and advancing numerous diseases (Yang et al., 2021; Ali and Kunugi 2020). In this study, we noted a substantial reduction in the O-GlcNAc modification of YTHDF2 during H_2_O_2_-induced oxidative stress conditions. This finding aligns with previous studies, where O-GlcNAc modification is widely recognized as a crucial mechanism in cellular stress responses (Fahie et al. 2022). It reinforces O-GlcNAc modification’s significance in the cellular response to external stressors.

**Fig. 11:**
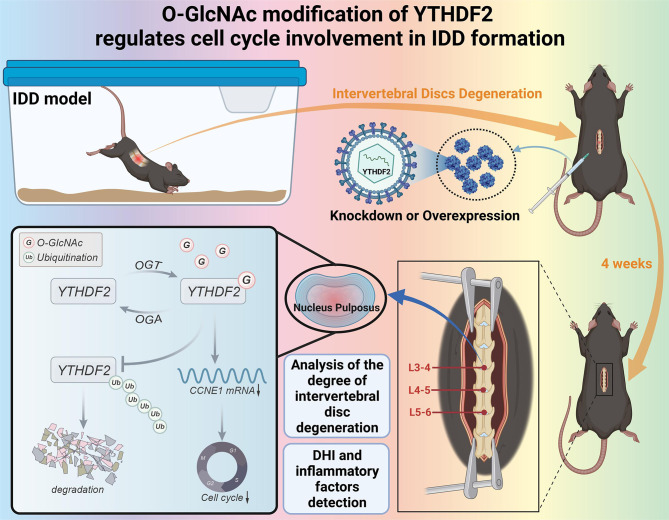
Molecular Mechanism of O-GlcNAc Modification of YTHDF2 in Regulating Cell Cycle Participation in IDD Formation

Intervertebral disc degeneration (IDD) is a cause of lower back pain and neck pain, greatly impacting the health and overall quality of life of the global population (Wu et al. 2023). This study primarily investigates the crucial role and function of the YTHDF2 gene and its O-GlcNAc in the development of IDD. Previous research has identified a correlation between the YTHDF2 gene and intellectual and developmental disorders (IDD) progression. The O-GlcNAcylation of YTHDF2 inhibits its ubiquitination, which reduces protein degradation and enhances the stability of downstream CCNE1 mRNA. As a result, it influences the cell cycle and contributes to the occurrence and development of IDD, as depicted in Fig. 11. The experimental results indicate that the overexpression of YTHDF2 and its modification through O-GlcNAc could alleviate the severity of IDD in mice. It offers novel insights into the mechanisms underlying IDD, providing potential therapeutic targets for its prevention and treatment.

This study presents new findings that elucidate the critical role of YTHDF2 and its O-GlcNAc in IDD, providing us with a comprehensive understanding of the pathogenesis of IDD. Our study of YTHDF2 has revealed its potential as a promising target for IDD treatment. Regulating YTHDF2 could offer novel treatment strategies for IDD.

While mice are frequently employed as model organisms in experimental research, it is important to acknowledge the inherent variations in their physiological and pathological mechanisms compared to humans. Hence, it is essential to validate the research findings using human samples. Intervertebral disc degeneration (IDD) pathogenesis may also involve the interaction of multiple genes and signaling pathways. Although YTHDF2 has shown its significance in this study, additional unidentified key factors may exist. Moreover, long-term clinical trials and research are still required to assess the therapeutic effects and long-term safety of regulating YTHDF2 in IDD.

## Electronic supplementary material

Below is the link to the electronic supplementary material.

Supplementary Material 1

Supplementary Material 2

Supplementary Material 3

Supplementary Material 4

Supplementary Material 5

## Data Availability

The original contributions presented in the study are included in the article/supplementary materials, further inquiries can be directed to the corresponding author.
