# Supplementary figures and images for: New perspectives on YTHDF2 O-GlcNAc modification in the pathogenesis of intervertebral disc degeneration
Source: Mol Med. 2024 Oct 18;30:180. doi: 10.1186/s10020-024-00876-x (PMC11488288; doi:10.1186/s10020-024-00876-x)

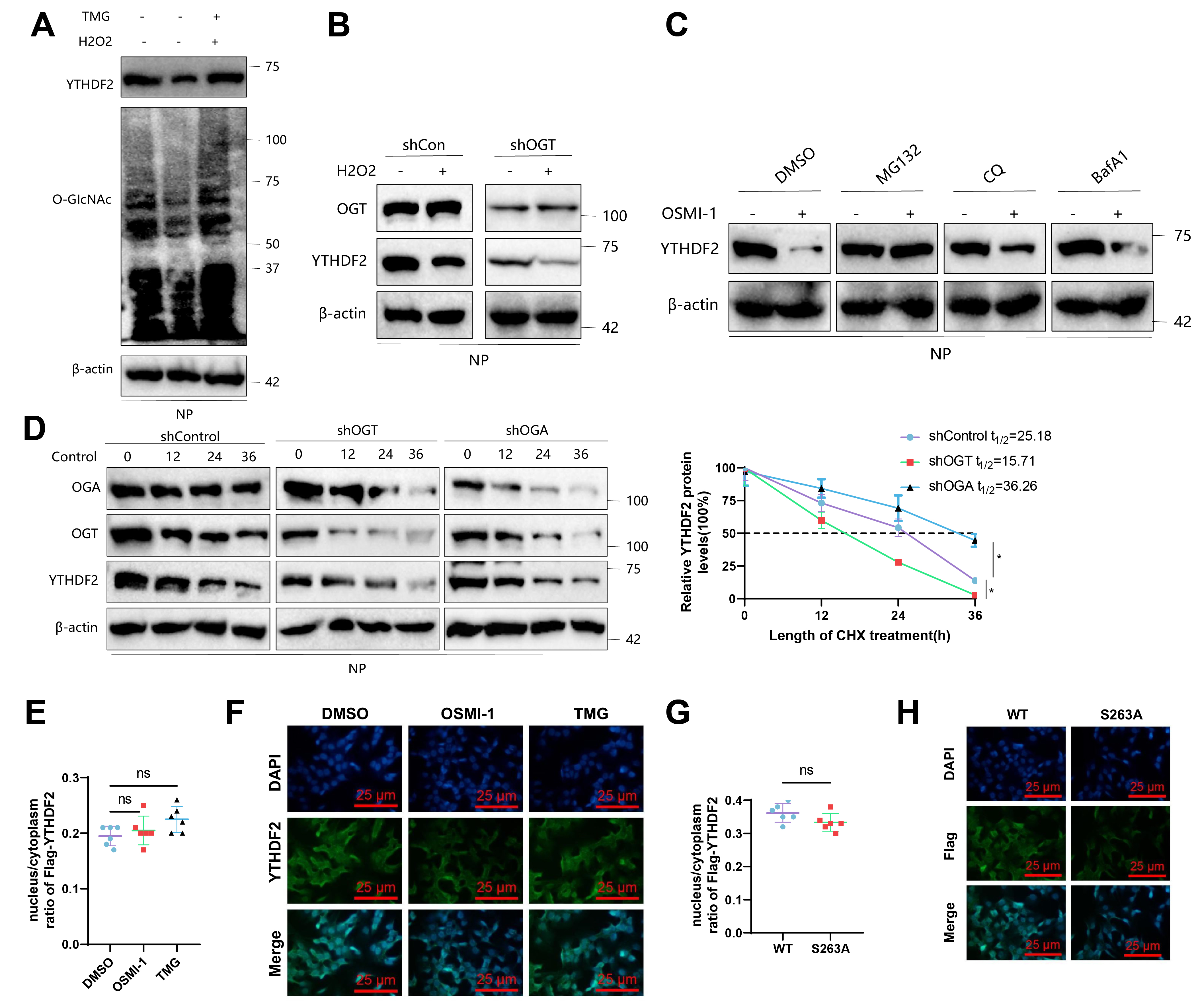

Supplement: Supplementary file 1 — Supplementary Material 1 [file 10020_2024_876_MOESM1_ESM.jpg]

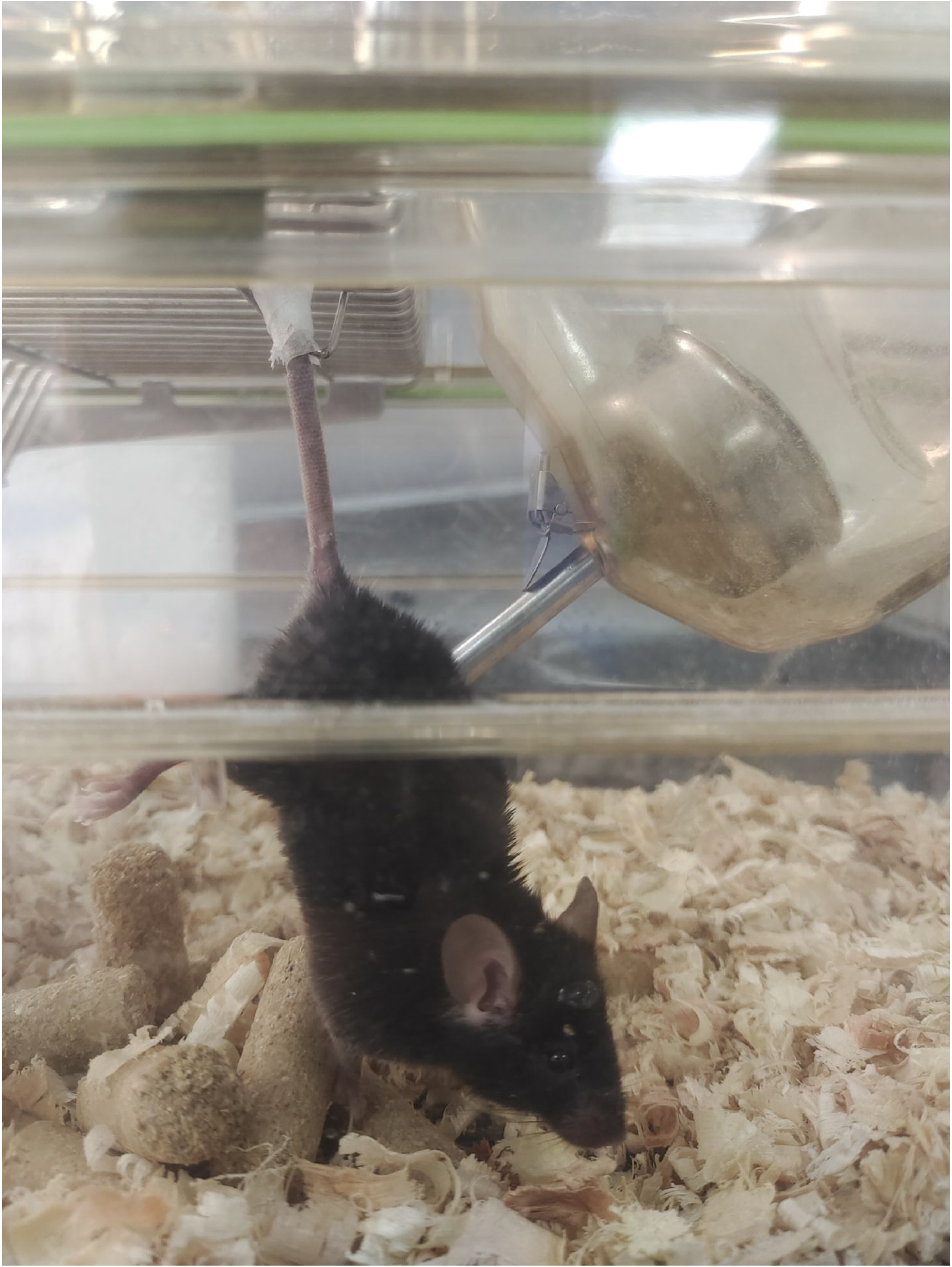

Supplement: Supplementary file 2 — Supplementary Material 2 [file 10020_2024_876_MOESM2_ESM.jpg]

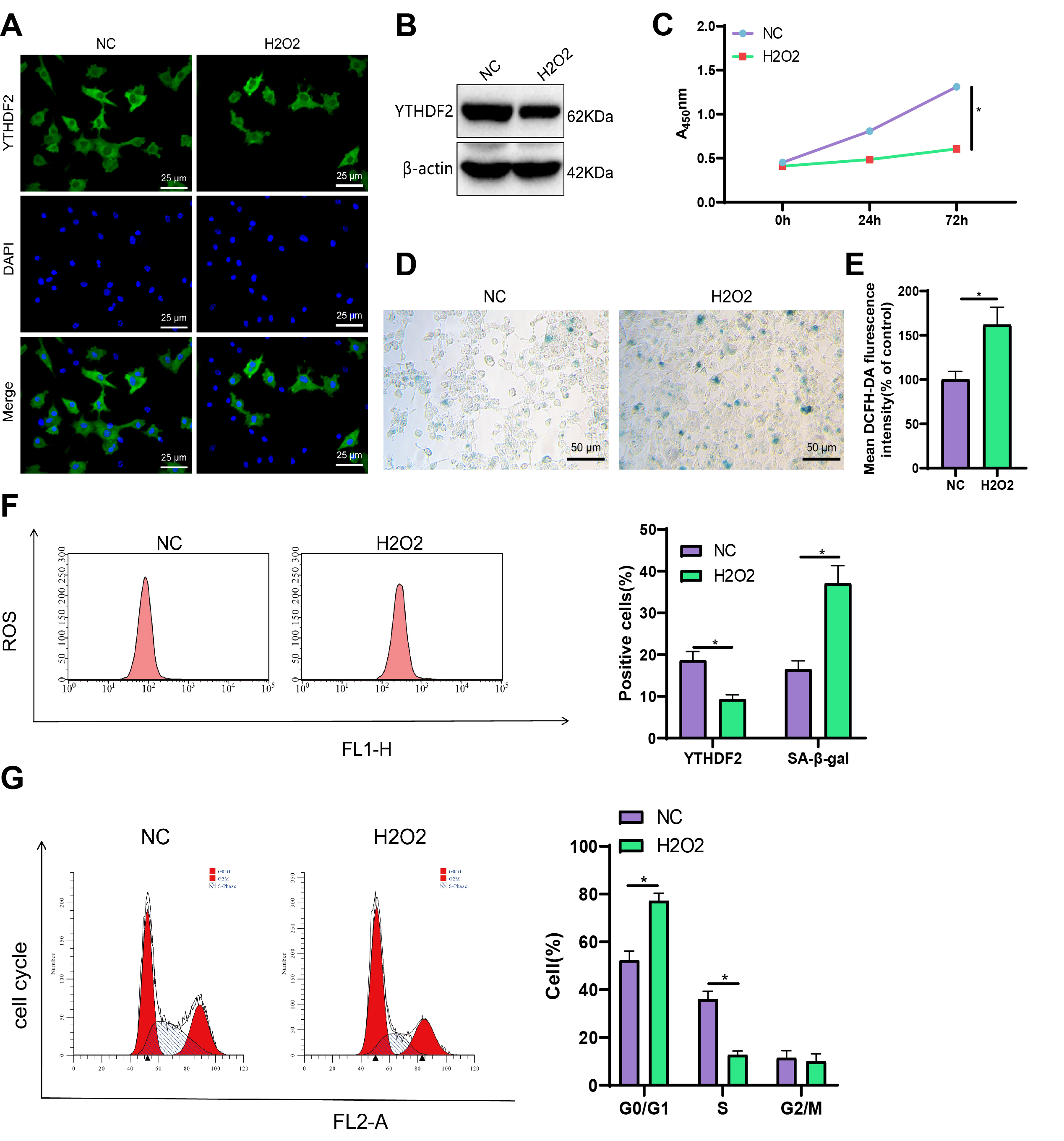

Supplement: Supplementary file 3 — Supplementary Material 3 [file 10020_2024_876_MOESM3_ESM.jpg]
