# Supplementary material for: New perspectives on YTHDF2 O-GlcNAc modification in the pathogenesis of intervertebral disc degeneration
Source: Mol Med. 2024 Oct 18;30:180. doi: 10.1186/s10020-024-00876-x (PMC11488288; doi:10.1186/s10020-024-00876-x)
Supplement: Supplementary file 4 — Supplementary Material 4 [file 10020_2024_876_MOESM4_ESM.docx]

**Table S1. qRT-PCR Primer sequence**

| Gene | Primer sequence |
| --- | --- |
| YTHDF2 | Forward: 5’-GAGCAGAGACCAAAAGGTCAAG-3’ |
| Verified | Reverse: 5’- CTGTGGGCTCAAGTAAGGTTC-3’ |
| MCM7 | Forward: 5’- AGTATGGGACCCAGTTGGTTC-3’ |
| Verified | Reverse: 5’- GCATTCTCGCAAATTGAGTCG-3’ |
| MCM5 | Forward: 5’-TGAACTCAAGCGGCATTACAA-3’ |
| Verified | Reverse: 5’- GGCTGTTTATGCAAGTGGTCA-3’ |
| ORC6 | Forward: 5’-ACTGCCGCACTACTCTCAG-3’ |
| Verified | Reverse: 5’-CACAGTCGATCAAGTATTGCCTT-3’ |
| ORC5 | Forward: 5’- CTTTGCAGTCCCTGTTTGGAG-3’ |
| Verified | Reverse: 5’-TGCGGCAGCTCTAAAGTTTTC-3’ |
| MCM3 | Forward: 5’-ACATGGCGGGCACAGTAG-3’ |
| Verified | Reverse: 5’- ACGCTGACAATCAGCCGATAC-3’ |
| ANAPC2 | Forward: 5’- TCCGATGACTGCGACTCTAGG-3’ |
| Verified | Reverse: 5’-CACTTCCACGAACCACTCCT-3’ |
| ANAPC5 | Forward: 5’- TTGACCGCCTGATTCTCACTG-3’ |
| Verified | Reverse: 5’-CAAAAGCTCTCGGTAACCCAAA-3’ |
| ANAPC1 | Forward: 5’-AAGAGCCCGAGAAACACCTG-3’ |
| Verified | Reverse: 5’- CCTCACAAGGCAGAGACTTGA-3’ |
| CDC16 | Forward: 5’- CATGAGCCTCTGACCACTTGT-3’ |
| Verified | Reverse: 5’-AATGACCGTTTCACTGGGCT-3’ |
| CCNE1 | Forward: 5’-GTTCCAAGCCCAAGTCCTGA-3’ |
| Verified | Reverse: 5’-GCTGACTGCTATCCTCGCTT-3’ |
| MCM6 | Forward: 5’- GAATCATTGGGGAGCGGTCA-3’ |
| Verified | Reverse: 5’- GAATGAAGCCGACAGGGTGA-3’ |
| β-actin | Forward: 5’- GTGACGTTGACATCCGTAAAGA-3’ |
| Verified | Reverse: 5’-GCCGGACTCATCGTACTCC-3’ |

**Table S2. Antibody Information**

| Antibody | Company | Cas | Dilution ratio/dosage |
| --- | --- | --- | --- |
| YTHDF2 | Abcam | ab246514 | 1:1000 |
| Collagen I | Abcam | ab260043 | 1:1000 |
| Collagen II | Abcam | ab34712 | 1:1000 |
| Collagen X | Abcam | ab182563 | 1:1000 |
| MMP-3 | Abcam | ab52915 | 1:1000 |
| MMP-13 | Abcam | ab39012 | 1:3000 |
| P19 | Abcam | ab80 | 1:1000 |
| P53 | Abcam | ab26 | 1:1000 |
| Sirt1 | Abcam | ab110304 | 1:1000 |
| SOD1 | Abcam | ab51254 | 1:50000 |
| SOD2 | Abcam | ab68155 | 1:1000 |
| CDK4 | Abcam | ab199728 | 1:1000 |
| CDK6 | Abcam | ab241554 | 1:1000 |
| CCNE1 | Abcam | ab211342 | 1:1000 |
| GAPDH | Abcam | ab181602 | 1:10000 |
| β-actin | Abcam | ab8226 | 1:1000 |
| O-GlcNAc | Thermofisher | MA1-072 | 1:1000 |
| OGA | Abcam | ab197389 | 1:1000 |
| OGT | Abcam | ab96718 | 1:500 |
| K48 | Abcam | ab140601 | 1:1000 |
| HA | Invitrogen | 26183 | 5 μg |
| Flag | Sigma-Aldrich | F3165 | 5 μg |
