# Supplementary material for: New perspectives on YTHDF2 O-GlcNAc modification in the pathogenesis of intervertebral disc degeneration
Source: Mol Med. 2024 Oct 18;30:180. doi: 10.1186/s10020-024-00876-x (PMC11488288; doi:10.1186/s10020-024-00876-x)
Supplement: Supplementary file 5 — Supplementary Material 5 [file 10020_2024_876_MOESM5_ESM.docx]

**Figure S1. Construction of Mouse IDD Model by Tail Suspension (TS) Method.**

**Figure S2: Effects of H_2_O_2_ on NP Cell Proliferation, Senescence, and Reactive Oxygen Species Levels.**

Note: (A) Immunofluorescence staining of NP cells under different treatment conditions (NC: no treatment; H_2_O_2_: treatment with 100 μM H_2_O_2_), showing YTHDF2 expression levels (green fluorescence) with DAPI staining indicating nuclear localization. Scale bar: 25 μm (200× magnification). (B) Western blot analysis of YTHDF2 expression levels under different treatment conditions. (C) CCK-8 assay measuring cell viability of NP cells from condition A. (D) SA-β-gal staining assessing cellular senescence under different treatment conditions. Scale bar: 50 μm (200× magnification). (E) Statistical analysis of the proportion of positive cells in images A and D. (F) Flow cytometry analysis of ROS levels in NP cells under different treatment conditions, with accompanying statistics. (G) Flow cytometry analysis of cell cycle distribution in NP cells under different treatment conditions. Data were analyzed using t-tests; * indicates a significant difference between the two groups (P < 0.05). Each cell experiment was performed with n=6 samples per group and repeated three times.

**Figure S3. Regulatory Role of O-GlcNAc Modification in YTHDF2 Protein Half-life and Subcellular Localization.**

Note: (A) Expression of YTHDF2 protein in NP cells treated with 25 μM TMG; (B) Protein expression of YTHDF2 after transfection with OGT shRNA lentivirus; (C) NP cells were treated with 10 μM MG132, 50 μM chloroquine (CQ), and 100 nM bafilomycin A1 (BafA1) for 6 h before treatment with 20 μM OSMI-1; (D) Analysis of the half-life and quantification of Flag-YTHDF2 in NP cells transfected with OGT, OGA, or control shRNA lentiviral vectors; (E-H) Subcellular localization of YTHDF2 determined by immunoblotting (E, G) or immunofluorescence staining (scale bar=25 μm, 200×) (F, H). Nuclei and cytoplasm were immunoblotted with anti-YTHDF2 or anti-Flag antibodies to measure the nuclear/cytoplasmic ratio of YTHDF2. NP cells were treated with 25 μM TMG or 20 μM OSMI-1 for 24 h (E, F), and NP cells were transfected with Flag-YTHDF2 (wild type or S263A). The expression levels at different time points were analyzed using a two-way analysis of variance, with independent sample t-tests conducted for pairwise group comparisons and a one-way analysis of variance for comparisons involving three groups. * indicates differences between groups (P < 0.05), experiment repeated 3 times.
